# Economic and technical analysis of hydrogen production and transport: a case study of Egypt

**DOI:** 10.1038/s41598-025-91589-6

**Published:** 2025-03-15

**Authors:** Marwa Ahmed Hassan, Noha Hany El-Amary

**Affiliations:** Electrical and Control Department, College of Engineering, Arab Academy for Science, Technology and Maritime Transport (AASTMT), Cairo, Egypt

**Keywords:** Hydrogen economy, Renewable energy, Hydrogen supply chain, Egypt, Technology integration, Environmental impact, Climate change

## Abstract

This study investigates the economic, technical, and logistical aspects of hydrogen production, with a particular focus on Egypt’s potential to emerge as a global hydrogen leader. The research is motivated by Egypt’s abundant renewable resources, strategic location, and increasing interest in hydrogen as a cornerstone of the energy transition. Using the Hydra simulation model developed in MATLAB/Simulink, the study evaluates the Levelized Cost of Hydrogen (LCOH) and Levelized Supply Costs of Hydrogen (LSCOH) across various scenarios, spanning from 2024 to 2050. These scenarios incorporate factors such as economic growth, technological advancements, energy policies, and infrastructure developments. Projections indicate that the hydrogen demand in Egypt is expected to reach 6.0 million tons by 2050, including both domestic consumption and export potential. Egypt’s low hydrogen production costs (4.5/kg) and strategic location position it to meet growing domestic demand while supporting exports. Comparative analyses with countries including France, Italy, Saudi Arabia, the UAE, Libya, and Jordan highlight Egypt’s competitive advantage, driven by its abundant solar and wind resources. The novel integration of LCOH and LSCOH methodologies, contextualized by Egypt’s renewable energy potential, fills a critical gap in the literature and offers a comprehensive framework for evaluating hydrogen production and trade competitiveness. The findings emphasize the importance of robust policy frameworks, including substantial incentives for renewable energy projects, de-risking mechanisms, and measures to reduce transport costs, in realizing Egypt’s hydrogen potential. Furthermore, the study explores the environmental and geopolitical implications of hydrogen production, underscoring Egypt’s strategic role in the global energy market. The results provide actionable insights for policymakers and industry stakeholders to position Egypt as a leader in hydrogen production, contributing significantly to the global energy transition.

## Introduction

This study aims to address these challenges by providing a comprehensive framework for evaluating the economic, logistical, and environmental aspects of hydrogen production, with a specific focus on Egypt and its comparative potential in the global hydrogen market. The introduction delves into the problem definition, a detailed literature review, while also addressing the study’s environmental impacts limitation and the paper’s contributions.

### Problem definition

The transition to renewable energy is a cornerstone for achieving global net zero emissions, with hydrogen playing a vital role due to its advantages in long-term storage and transportability. However, the economic feasibility of hydrogen production, particularly in off-grid settings, presents significant challenges. A primary concern is the Levelized Cost of Hydrogen (LCOH), which varies widely depending on geographic location and the renewable energy source used, such as photovoltaic (PV) systems or wind turbines. Understanding these cost variations is crucial for evaluating the economic viability of hydrogen production projects and ensuring their competitiveness. A major limitation in current evaluations lies in the reliance on static interest rates that fail to account for country-specific investment risks. This can lead to inaccurate LCOH calculations and obscure the true economic picture. To address this, a more nuanced financial analysis is necessary-one that incorporates regional economic conditions and investment risks. Key financial parameters, such as the imputed interest rate (IIR) and the country-specific Weighted Average Cost of Capital (WACC), significantly influence LCOH and must be accurately assessed to evaluate the cost-competitiveness of hydrogen production effectively. In addition to production costs, the cost of transporting hydrogen, whether via pipelines or shipping, is another critical factor. These transport costs, when combined with WACC, heavily impact the overall cost of delivering hydrogen to end-users, especially in target regions. Evaluating the competitiveness of imported hydrogen requires a holistic understanding of these transport costs. Addressing these financial and logistical challenges calls for robust policy frameworks that incentivize investment, streamline regulatory processes, and foster international collaborations to enhance hydrogen production and trade. Moreover, comparing hydrogen production sites and scaling options for electrolyser plants is a complex endeavor, influenced by diverse technical and economic factors and the recent volatility in energy markets. This complexity highlights the need for a thorough and systematic approach to managing investment risks and economic uncertainties. A comprehensive evaluation framework is essential for making informed decisions regarding hydrogen production and its economic feasibility across various locations.

### Literature review

Hydrogen technologies have emerged as a cornerstone for achieving global low-carbon energy transitions due to their potential to integrate renewable energy sources and enhance grid stability. McPherson et al.^1^ emphasize hydrogen’s ability to enable up to 20% renewable energy penetration by acting as a buffer during periods of variability. However, their study is theoretical, lacking insights into practical challenges such as cost dynamics and infrastructure scalability. Similarly, Kovač et al.^2^ highlight hydrogen’s role in replacing fossil fuels, with production costs ranging between 2*and*8 per kg depending on the technology and region. Despite these insights, the study fails to address the economic barriers faced by developing economies in adopting hydrogen technologies. Robles et al.^3^ explore hydrogen’s integration into energy supply chains, identifying transport costs of up to 1.5 per kg in remote regions. However, they do not propose solutions to mitigate these logistical challenges or reduce transportation costs. Aba et al.^4^ compare hydrogen and electricity as energy carriers, emphasizing hydrogen’s energy density, which is three times that of fossil fuels. Despite this advantage, they neglect storage costs, which often exceed 30% of the total system expenses. Jamal et al.^5^ report a 20% efficiency improvement in hydrogen fuel cell technology over the past decade, underscoring technological advancements. However, economic evaluations ensuring their long-term viability are lacking. El-Emam and Özcan^6^ highlight high-temperature electrolysis, estimating cost reductions of up to 25%. However, the study overlooks regional variability in production potential and the financial barriers to deploying this technology in underdeveloped regions. Frieden and Leker^7^ present a quantitative review of hydrogen cost trends, reporting a 15% reduction in levelized costs over the past decade. However, they fail to consider regional policies and market conditions, which often create cost discrepancies of up to 3 per kg. Lee et al.^8^ evaluate hydrogen production costs in Korea, demonstrating potential reductions of 10%, but their analysis does not address how these findings could be extrapolated to regions with different economic structures or renewable resource availability. Kamran and Turzyński^9^ review hydrogen systems comprehensively, highlighting operational cost variations of up to 25% based on infrastructure and policies. Despite this, they do not propose strategies to address these variations. Rezaei et al.^10^ focus on co-production of electricity and hydrogen from wind, identifying transport costs that increase by $1-$2 per kg in off-grid locations. However, they do not propose solutions for reducing these costs. Ates and Calik^11^ address public awareness, finding only 40% of populations in developing regions are familiar with hydrogen’s benefits. Their study fails to suggest ways to overcome this barrier, leaving a gap in practical adoption strategies. Fakhreddine et al.^12^ note a cost gap of $0.5-$1 per km between hydrogen and electric vehicles, highlighting scalability challenges in hydrogen fuel cell technologies. However, a roadmap for reducing these costs is missing. Ratnakar et al.^13^ discuss hydrogen supply chain challenges, identifying 20% cost variations based on carrier types, but do not provide specific recommendations for mitigating these variations. Mazloomi and Gomes^14^ provide a broad overview of hydrogen as an energy carrier but fail to quantify the economic benefits of modular electrolyzers, which are gaining traction in the industry. Rasul et al.^15^ report solar-driven electrolysis achieves only 80% efficiency under optimal conditions. However, technological improvements required to enhance efficiency and reduce costs are not addressed. Ishimoto et al.^16^ analyze hydrogen transport from Norway, noting off-grid transport costs add 35% to total expenses. However, alternative transport methods to mitigate these costs are unexplored. Abdalla et al.^17^ and Faye et al.^18^ emphasize the potential of advanced materials to reduce storage costs by 18%. Yet, scalability and long-term durability in real-world conditions remain unaddressed. Navas-Anguita et al.^19^ assess hydrogen production for road transport, failing to capture off-grid cost increases of 2 per kg. Ishaq et al.^20^ discuss regional cost variations of up to 30% but do not propose strategies for managing these differences. Maggio et al.^21^ explore hydrogen’s market impact but fail to address fluctuating energy prices, which can influence costs by 15%. Esily et al.^22^ estimate that Egypt’s hydrogen production costs are 20% higher than global averages, but solutions to enhance cost competitiveness in local contexts are absent. Nasser et al.^23^ and Youssef et al.^24^ focus on hybrid renewable systems and hydrogen storage in Upper Egypt, respectively. Both studies identify logistical challenges that increase costs by 1.5 per kg but fail to offer practical solutions. Salama et al.^25^ propose control strategies for hydrogen production, showing a 15% efficiency improvement but not addressing regional economic factors. Alhussan et al.^26^ explore economies of scale in green hydrogen production, identifying potential cost reductions of 20%. However, they overlook the technological and policy barriers to achieving these economies of scale. Mahmoud et al.^27^ review geothermal hydrogen production, noting potential cost reductions of 0.5 per kg. Yet, their study does not address the high capital investments required for geothermal projects. Ismail et al.^28^ and Jang et al.^29^ discuss photovoltaic hydrogen production, highlighting efficiency gains of up to 30%. However, both studies neglect broader economic implications, such as high initial investments. Fragiacomo and Genovese^30^ analyze scalable hydrogen production but fail to address cost variations across different geographic contexts. Nicita et al.^31^ evaluate photovoltaic electrolysis, neglecting scaling challenges critical for commercial viability. Kakoulaki et al.^32^ report cost discrepancies of up to 3 per kg but do not propose solutions for cost optimization. Gerloff^33^ focuses on hydrogen production in Germany, offering limited insights for other regions. Müller and Eichhammer^34^ discuss economic complexity but omit regional variations affecting costs by 25%. Benalcazar and Komorowska^35^ explore green hydrogen in Poland but fail to address infrastructure gaps and market readiness. He et al.^36^ discuss biomass-based hydrogen production, emphasizing cost impacts of 15%, but neglecting regional challenges. Raza et al.^37–43^ provide detailed analyses on hydrogen adoption in Pakistan. Raza et al.^37^ propose a climate policy simulation model, demonstrating the potential for renewable integration to reduce emissions by 35%. However, the reliance on static assumptions regarding market conditions and renewable energy potential limits the applicability of the model in dynamic energy markets. Raza et al.^38^ emphasize the role of hydrogen in reducing energy import dependency by 25%, showcasing its potential to enhance energy security. Despite these findings, the scalability of such systems in underdeveloped regions with limited infrastructure is not addressed. Similarly, Raza et al.^39^ investigate strategies for transitioning from fossilized to defossilized energy systems, with hydrogen as a bridging technology. They estimate a potential 30% reduction in fossil fuel reliance, yet the study does not fully explore the financial and technical challenges of implementing such transitions. Raza et al.^40^ report a 20% efficiency improvement in smart grid systems using hydrogen, highlighting operational advantages. However, the lack of detailed economic analyses for implementing smart grids in resource-constrained regions remains a gap. Raza et al.^41^ analyze smart grid infrastructure strategies, demonstrating potential operational cost reductions of 15%. Nevertheless, they fail to address the applicability of these strategies in off-grid settings or regions with outdated infrastructure. Raza et al.^42^ identify policy-relevant insights, showing a 25% improvement in energy reliability through smart grid innovations. However, they do not propose strategies for regions lacking policy support for hydrogen adoption. Finally, Raza et al.^43^ discuss hydrogen storage, highlighting potential cost reductions of 18% with advanced materials. Yet, challenges such as material durability and scalability in real-world conditions remain unaddressed. Recent studies from 2023 to 2024 provide critical insights into hydrogen technologies. Odenweller and Ueckerdt^44^ highlight a significant gap between global targets for green hydrogen deployment and actual implementation, emphasizing the need for realistic policy frameworks. However, they do not provide solutions to address technological barriers, such as electrolyzer efficiency or storage limitations. Hordvei et al.^45^ estimate that Europe requires ${\EUR \text{\euro } }$180 billion in investments by 2030 to meet green hydrogen production targets. While they provide a clear picture of financial requirements, their focus on Europe limits the applicability of findings to other regions with different economic conditions. Kim et al.^46^ investigate the economic synergy of integrating direct air capture systems with green hydrogen production, demonstrating potential cost reductions of 20%. However, scalability challenges in deploying this technology at a global level remain unaddressed. Goodarzi and Li^47^ evaluate the energy-water-hydrogen nexus, identifying regional variations in resource availability as a key factor in hydrogen production costs. Despite this, they do not propose solutions to harmonize these variations or improve resource efficiency. Sebbagh et al.^48^ highlight the role of green hydrogen in sustainable energy transitions, estimating production cost reductions of up to 30% with advanced technologies. However, their study does not explore the infrastructure and regulatory challenges required to achieve these reductions. Holmes-Gentle et al.^49^ present advancements in solar-to-hydrogen conversion technologies, achieving efficiency gains of up to 15%. Nevertheless, their system remains experimental, raising concerns about its commercial viability. Fehr et al.^50^ report a record solar-to-hydrogen efficiency of 20.8% using integrated halide perovskite photoelectrochemical cells. While this represents a significant technological breakthrough, the study fails to address material stability issues, which hinder widespread adoption. While prior studies have extensively analyzed hydrogen technologies, several critical gaps persist, including regional cost variability, scalability challenges, and public engagement strategies. Building on recent advancements, this study employs advanced modeling techniques and detailed regional data to bridge these gaps. Our approach offers a comprehensive framework that evaluates economic and logistical constraints while proposing region-specific policy and technological solutions. These contributions aim to enhance the scalability and cost-effectiveness of hydrogen technologies, particularly in underrepresented regions like developing economies.Additionally, this study will evaluate the environmental impacts of hydrogen production, with a focus on carbon emissions and resource sustainability, providing a holistic view of its viability.

### Paper contribution

This paper advances renewable hydrogen production and economic evaluation, emphasizing off-grid solutions and hydrogen trade dynamics. It introduces an innovative simulation-based methodology to assess the Levelized Cost of Hydrogen (LCOH) and Levelized Supply Cost of Hydrogen (LSCOH), optimizing electrolyzer sizing across Egypt, Libya, Saudi Arabia, Jordan, UAE, Italy, and France. By integrating economic growth projections, energy policies, technological advancements, and transport infrastructure, the study ensures a realistic and forward-looking analysis.

A key contribution is the detailed financial analysis of imputed interest rates (IIR) and Weighted Average Cost of Capital (WACC), particularly highlighting the impact of WACC on LCOH in emerging markets like Egypt. This extends prior research by incorporating dynamic, region-specific financial factors often overlooked in earlier models. Additionally, the paper evaluates hydrogen’s competitiveness in production and importation by calculating LSCOH, providing a unified framework for understanding total supply chain costs and enabling cross-country comparisons.

Focusing on Egypt’s potential as a hydrogen leader, the study examines its production capacity, export potential, and the impact of financial and logistical factors. While Egypt’s abundant solar and wind resources provide a competitive advantage, challenges such as infrastructure gaps, high WACC, and water scarcity could limit scalability. Practical recommendations are offered to leverage Egypt’s renewable resources and infrastructure to strengthen its global market position.

Despite its comprehensive insights, the study acknowledges limitations such as data availability, modeling assumptions, and potential technological advancements (e.g., next-generation electrolyzers, Liquid Organic Hydrogen Carriers (LOHC), and advanced energy storage). Future research could explore emerging hydrogen transport solutions, including LOHC and alternative shipping routes. The paper highlights the role of robust policy frameworks, emphasizing subsidies, tax incentives, and government-backed guarantees to address financial and infrastructure barriers. Egypt’s evolving policies require stronger incentives to compete with proactive investments in the EU and Middle East. Regional and international collaboration, particularly in MENA and with European hydrogen importers, could optimize infrastructure, enhance resource sharing, and drive hydrogen market growth. Environmental sustainability is a core focus, advocating for renewable energy integration, carbon pricing, and lifecycle assessments to align hydrogen production with climate goals. Addressing local challenges, particularly water scarcity for electrolysis, through desalination and wastewater reuse is also emphasized. Ultimately, this study provides a roadmap for Egypt’s transition to a sustainable hydrogen economy, leveraging its renewable resources, geographic advantages, and policy evolution. By offering actionable insights for hydrogen adoption across diverse contexts, the research lays the foundation for future studies on technological innovation and policy frameworks driving the global energy transition.

### Paper structure

The paper is organized as follows: Section One introduces the study, outlining its objectives and setting the context for the research. Section Two details the methodology, focusing on the Hydra simulation model used to analyze hydrogen production costs. Section Three discusses Hydrogen Levelized Costs Based on Plant Size and Location, while Section Four examines Transport and Infrastructure Costs for Hydrogen Carriers. Section Five explores Policy Implications and Technological Advancements in hydrogen production. Section Six concludes the paper, summarizing the findings and highlighting areas for future research.

### Theoretical framework

This study employs a comprehensive model to assess the Levelized Cost of Hydrogen (LCOH) and Levelized Supply Cost of Hydrogen (LSCOH), integrating economic, technical, logistical, and policy-related factors. Designed to capture region-specific nuances, the model overcomes limitations of static approaches by incorporating renewable energy variability, financial risks, and transport complexities. Unlike previous studies that assume uniform conditions, this dynamic approach allows for a more accurate cost assessment tailored to each region, including Egypt, where renewable resources and infrastructure pose unique challenges. The model’s theoretical foundation highlights the necessity of multidimensional analysis in hydrogen economics, considering renewable resource availability, investment climate, infrastructure, and policy environments. Economic factors such as the Weighted Average Cost of Capital (WACC) and imputed interest rates (IIR) are integrated to reflect regional investment risks, particularly in developing markets like Egypt. Unlike prior studies, which overlook these financial barriers, this model ensures investment challenges are accounted for in cost calculations. Technical factors, including renewable energy systems like photovoltaics and wind, are incorporated using reliable datasets from PVGIS and the Global Wind Atlas. These datasets capture regional solar and wind potential, improving the accuracy of hydrogen production simulations. Logistical aspects, particularly hydrogen’s low energy density and the need for specialized transport infrastructure (pipelines, liquefaction, ammonia conversion), are also addressed. Given Egypt’s infrastructure gaps, transport costs play a critical role in determining overall hydrogen export feasibility. Policy dynamics are central to the model, incorporating government interventions such as subsidies, tax incentives, and regulatory frameworks. Drawing from Kamran and Turzyński^9^, this integration reflects how policy shapes technological adoption and market competitiveness. LCOH measures baseline production costs, while LSCOH extends the analysis to include transport, ensuring a complete cost evaluation from production to delivery. This unified approach improves the accuracy of cost estimations, as demonstrated in studies by Robles et al.^3^ and Ishimoto et al.^16^. By dynamically integrating region-specific financial risks, renewable energy variability, transport costs, and policy factors, this model advances hydrogen economics research, providing a holistic evaluation of production feasibility. Its flexibility and regional specificity offer valuable insights for policymakers and industry stakeholders navigating the complexities of the global hydrogen economy, particularly in emerging markets like Egypt.

## Methodology

**Fig. 1:**
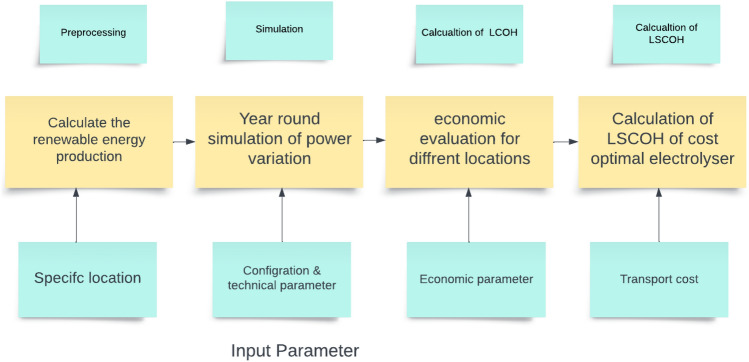
The study employed the Hydra model, selecting a specific methodological approach and input parameters.

The methodology employs the Hydra simulation model, developed in MATLAB/Simulink, which comprehensively represents the components of renewable energy generation and hydrogen production through electrolysis. The use of this model is justified by its ability to incorporate diverse variables-economic, technical, and logistical-providing a dynamic framework for evaluating hydrogen production and its competitiveness across different regions and time frames. Data for the study were obtained from reliable sources, including the PVGIS, RENEWABLES, and GLOBAL WIND ATLAS platforms, alongside national energy reports, policy documents, and academic literature, ensuring comprehensive and up-to-date input for the model. Initially, renewable power generation profiles were meticulously generated based on the chosen geographic location, as depicted in Fig. 1. These profiles were integrated into the model to evaluate renewable resource availability and assess its impact on hydrogen production costs. The efficiency of the electrolysis system is assumed to be 70% based on typical commercial electrolyzers available in the market. Renewable energy availability is assumed to be based on historical data from the PVGIS and GLOBAL WIND ATLAS platforms, with solar irradiance values specific to the location (e.g., 2100 kWh/$\hbox {m}^2$/year for Egypt) and wind speed values aligned with regional data (e.g., average wind speed of 6.5 m/s). It is assumed that the energy generated from renewable sources is utilized directly for hydrogen production, with no significant energy curtailment or grid integration issues. In case of excess energy, it is assumed to be dissipated and not stored or fed back into the grid. The WACC for each region is derived based on country-specific risk factors, with a higher value assumed for Egypt due to its developing market status. Other countries considered in this study, such as Saudi Arabia, the UAE, France, Italy, Libya, and Jordan, have their WACC values adjusted according to their respective economic conditions and financial markets. The optimization algorithm used for matching electrolysis power with peak renewable energy power is based on a simplified model that assumes optimal matching without significant technical limitations on the electrolysis system’s response time or operational flexibility. The electrolysis system operates in an off-grid or island mode, ensuring that any surplus energy not utilized for hydrogen production is dissipated rather than stored or fed back into the grid. By using the calculated renewable power profile specific to the chosen location, the potential for hydrogen production and the utilized electrical energy were analyzed by systematically varying the nominal electrolysis power. This analysis accounted for the technical parameters of the electrolysis system, including efficiency, response time, and operational limits. In the post-processing phase, an optimization algorithm was employed to identify the cost-optimal pairing of electrolysis power and the peak power of the renewable source. This optimization was based on the Levelized Cost of Hydrogen (LCOH), which considers both capital and operational expenses. Finally, the transportation costs for various hydrogen transport routes to Egypt were incorporated into the model. These routes included different modes of transport such as pipelines, shipping, and road transport, each with associated costs and efficiencies. The Levelized Supply Cost of Hydrogen (LSCOH) was then calculated for the most cost-effective hydrogen production scenario at the specified location. To extend the analysis, the methodology incorporates a dedicated simulation approach for modeling decentralization and automation trends in hydrogen production. Historical data on hydrogen consumption rates, renewable energy potential, and country-specific WACC values were sourced from financial market reports, technological datasets, and academic publications. Decentralization was modeled as a shift toward localized and self-sufficient energy systems, enhancing flexibility and resilience, while automation was represented through advanced technologies integrated to streamline operations and improve efficiency. Renewable energy variability was analyzed using solar irradiance and wind speed data specific to Egypt and comparative regions. Policy scenarios (Policy A, B, and C) were designed to evaluate their impacts on investment and technological adoption, incorporating parameters such as government support levels and regulatory frameworks. This enhanced methodology offers nuanced insights into the interplay of economic, technical, and policy dynamics, providing actionable recommendations for stakeholders.

### Modeling decentralization and automation trends

Decentralization was modeled as a transition toward localized and self-sufficient energy systems. The model incorporated grid independence as a key factor, ensuring that energy produced is utilized locally to enhance resilience against outages. The optimization algorithms considered factors like localized renewable energy variability and storage capacity to simulate how decentralized systems could adapt to regional demand profiles and weather conditions. Automation was represented through the integration of advanced technologies, such as automated control systems and AI-based operational tools. These technologies were modeled to reduce human error, streamline operations, and enhance overall efficiency. For instance, the model accounted for faster response times in energy dispatch and predictive maintenance enabled by automation. The interplay between decentralization and automation was incorporated into the Hydra simulation by varying the degree of localized energy usage and the level of technological adoption. These variations allowed the study to simulate multiple scenarios, ranging from traditional centralized systems to fully automated decentralized networks, capturing the spectrum of potential future energy infrastructures.More detais found in Table 1.

**Table 1:** Illustrative parameters for decentralization and automation modeling.

Parameter	Decentralization impact	Automation impact
Local energy utilization (%)	85%	N/A
Resilience score	increased to 90%	N/A
Operational efficiency	N/A	Improved by 25%
Maintenance costs ($/kWh)	N/A	Reduced by 20%

### Estimation of regional renewable energy generation

Hourly renewable generation profiles for various locations were meticulously created as the foundation for the simulation. The photovoltaic (PV) profiles were derived from hourly radiation data collected over one year, sourced from the PVGIS database^51^. This data was then processed using advanced algorithms to account for local climatic conditions and seasonal variations, ensuring high accuracy in the generation profiles. For wind energy, hourly wind profiles were calculated using the logarithmic height formula and normalized power curves from different turbine classes. This calculation was based on one year of wind speed data from PVGIS. To enhance the accuracy of the wind power profiles, they were scaled to match the International Renewable Energy Agency (IRENA) database^51^ values for the respective country as of 2020. This scaling process involved correcting for discrepancies and biases in the publicly available wind data, thereby ensuring that the profiles accurately reflected real-world conditions. The detailed methodology for generating and calculating the input profiles for the simulation, including data processing techniques and validation steps, is comprehensively described in the Annex. This approach ensures that the simulation model is grounded in robust, empirically validated data, providing reliable insights into the potential for renewable energy generation and hydrogen production in the chosen locations.

### Determination of country-specific risk premiums in WACC

**Fig. 2:**
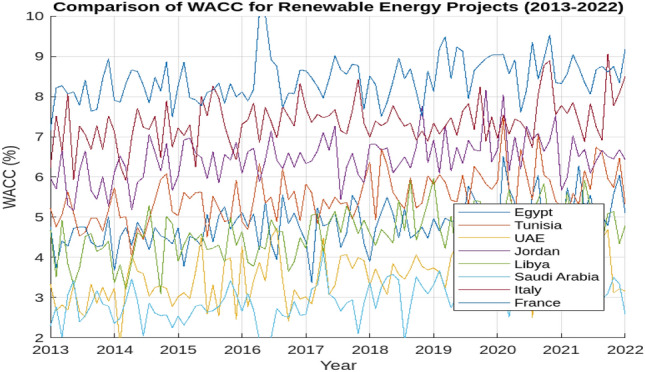
Comparison of WCAA projiect for the previous year.

The Weighted Average Cost of Capital (WACC) is a crucial metric for evaluating the average return on investments and assessing the risk profile of companies investing in various regions^52^. Essentially, an increasing WACC indicates higher investment risk. In this study, we determined the WACC for an Egypt-based company investing in hydrogen projects across different global locations. This enables a comparison of the Levelized Cost of Hydrogen (LCOH) in various regions, incorporating country-specific risks. Figure 2 presents the WACC results from 2013 to 2022 for the countries considered in this paper. Notably, the WACC in 2022 is approximately double that of 2021. Investments in Egypt or Libya are significantly more expensive than in Saudi Arabia, Jordan, or the United Arab Emirates due to higher Equity Risk Premiums (ERP), reflecting the increased risk associated with equity investments in power generation. Currently, renewable energy projects in Saudi Arabia and Jordan have similar capital costs, while the United Arab Emirates is the most cost-effective location regarding WACC. The detailed methodology and results of the WACC calculations are provided in the Annex. For subsequent analyses, we evaluated the LCOH based on the lowest WACC (low-interest phase—2021) and the latest WACC (high-interest phase—2022) for the selected locations to assess the impact of WACC on LCOH and the associated risks for each location.

### Simulation model

To calculate hydrogen production, the simulation model *HYDRA* was employed, as outlined in Refs.^41–45^. These sources detail the process for determining the hydrogen output based on the available electrical power and the electrolysis system’s specifications. The technical parameters for the proton exchange membrane (PEM) electrolysis system are summarized in Table 2. In this study, the renewable generation power $R_{\text {res}}(t)$ was used as input to determine the quantity of hydrogen produced. When the available renewable power $R_{\text {res}}(t)$ is below the minimum required power $R_{\text {ely}}^{\text {min}}$ for the electrolysis system, no hydrogen is produced. When the renewable power is between the minimum and maximum electrolysis power $R_{\text {ely}}^{\text {max}}$, it is utilized for hydrogen production, considering the power gradients $dR_{\text {ely}}$. If the renewable power exceeds the maximum capacity of the electrolyser, the excess power is dissipated as $R_{\text {dis}}(t)$. Our study focuses on PEM electrolysers due to their efficient response to fluctuations in renewable power. The specified output pressure of 30 bar facilitates easier downstream mechanical compression, which is typical for PEM systems.

Since the system operates off-grid, surplus energy cannot be fed into the grid or otherwise utilized. The utilization factor $u_{\text {util}}$, defined by the following equation, represents the ratio of energy used for hydrogen production $T_{\text {ely}}$ to the total renewable energy produced $T_{\text {res}}$:

$$\begin{aligned} u_{\text {util}} = \frac{T_{\text {ely}}}{T_{\text {res}}} = \frac{\int _{0}^{365} R_{\text {ely}}(t) \, dt}{\int _{0}^{365} R_{\text {res}}(t) \, dt} = \frac{T_{\text {res}} - T_{\text {dis}}}{T_{\text {res}}} \end{aligned}$$

where $R_{\text {ely}}(t)$ represents the power used for electrolysis at time $t$, $R_{\text {res}}(t)$ denotes the renewable power available at time $t$, $T_{\text {ely}}$ is the total energy used for hydrogen production, $T_{\text {res}}$ is the total energy produced by the renewable energy system, and $T_{\text {dis}}$ refers to the energy dissipated.

**Table 2:** Technical characteristics of the PEM electrolysis system.

Characteristic	Value
Maximum efficiency at optimal point	65%
Efficiency at nominal operation	58%
Hydrogen production pressure	25 bar
Positive/negative power gradient	$\pm 8$ % of $R_{\text {ely}}$/s
Minimum electrolysis power	4% of $R_{\text {ely}}$
Operating temperature	75 $^\circ$C

### Financial assessment through levelized hydrogen costs

To assess the financial feasibility and competitiveness of hydrogen production systems, a thorough economic evaluation is essential. This process involves calculating the net present values (NPV$^*$) of various cost components, providing a detailed insight into the financial aspects of hydrogen production. These calculations are crucial for comparing different production scenarios and investment strategies by accounting for both capital and operational expenditures, as well as considering economic factors like interest rates and price escalations. The economic parameters used in the LCOH and LCOE calculations are outlined in Table 3.

The first step is to calculate the net present value of capital expenditures (NPV$^*_{\text {CapEx}}$), which represents the total initial investment required to establish the hydrogen production infrastructure. Additionally, the net present value of operational expenditures (NPV$^*_{\text {OpEx}}$) encompasses all ongoing costs associated with the production process. These NPV$^*$ values are critical for determining the levelized cost of hydrogen (LCOH), which represents the average cost per unit of hydrogen over the entire project lifespan.

To account for the time value of money, the levelized cost of hydrogen is calculated using the imputed interest rate (IIR), which discounts future cash flows to their present value. In this calculation, the quantity of hydrogen produced in year $n$ is denoted by $m_{H_2,n}$, while $\text {hpr}$ represents the rate at which hydrogen prices increase over time. The evaluation is conducted over a total period represented by $N$. The equation for calculating LCOH with IIR is given by:

1 $$\begin{aligned} LCOH_{IIR} = \frac{\text {NPV}^*_{\text {CapEx}} + \text {NPV}^*_{\text {OpEx}}}{\sum _{n=0}^{N} m_{H_2,n} \cdot \left( \frac{1 + \text {hpr}}{1 + \text {IIR}}\right) ^n} \end{aligned}$$

This approach allows for the assessment of hydrogen production costs adjusted for anticipated price increases and the time value of money.

When the Weighted Average Cost of Capital (WACC) is used instead of IIR, the calculation is adjusted to reflect the specific financial environment of the production location. Here, $\text {WACC}$ replaces $\text {IIR}$ as the discount rate, and the price increase rate ($\text {pr}$) is set to zero. The LCOH using WACC is calculated as:

2 $$\begin{aligned} LCOH_{WACC} = \frac{\text {NPV}^*_{\text {CapEx}} + \text {NPV}^*_{\text {OpEx}}}{\sum _{n=0}^{N} m_{H_2,n} \cdot \left( \frac{1 }{1 + \text {WACC}}\right) ^n} \end{aligned}$$

This approach offers a financial viewpoint by factoring in the cost of capital, making it valuable for evaluating various investment options.

The levelized supply cost of hydrogen (LSCOH) incorporates not just the LCOH but also the expenses associated with transporting hydrogen from the production site to the point of demand. This can be expressed as:

3 $$\begin{aligned} LSCOH_{WACC} = LCOH_{WACC} + P_{\text {Trans}} \end{aligned}$$

where $P_{\text {Trans}}$ denotes the additional cost associated with transportation, providing a comprehensive measure of the total supply cost. In the context of energy procurement, the levelized cost of energy (LCOE) is determined by evaluating the net present value of capital expenditures ($\text {NPV}^*_{\text {CapEx, RES}}$) and operational expenditures ($\text {NPV}^*_{\text {OpEx, RES}}$) for renewable energy systems. In this calculation, $E_{\text {Ely}}$ denotes the energy produced from renewable sources, while $\text {pr}$ represents the general price increase rate. The imputed interest rate (IIR) is employed to discount future energy costs. The formula for LCOE is:

4 $$\begin{aligned} LCOE = \frac{\text {NPV}^*_{\text {CapEx, RES}} + \text {NPV}^*_{\text {OpEx, RES}}}{\sum _{n=0}^{N} E_{\text {Ely}} \cdot \left( \frac{1 + \text {pr}}{1 + \text {IIR}}\right) ^n} \end{aligned}$$

This calculation provides a measure of the cost per unit of energy generated, integrating both initial and ongoing costs over the evaluation period. By employing these methods, the economic evaluation captures the full spectrum of costs associated with hydrogen production and renewable energy systems, enabling informed decision-making and investment strategies.

**Table 3:** Economic parameters for hydrogen production (in USD).

General	Electrolysis
Interest rate on investment-IIR	4–6%
Weighted average cost of capital-WACC	3.0–7.0%
Evaluation period-$$N$$	20 years
Rate of price increase-$$pr$$	4.0%
Rate of hydrogen price increase-$$hpr$$	4.0%
Installation expenses (relative to initial electrolyser investment)	60%
Electrolysis	
Rate of price increase	$$-4.0$$%
Ongoing operational costs (relative to initial investment)	6%
Initial investment for systems over 10 MW	$ 1188/kWp
Costs for replacement (stack)	$ 540/kWp
Stack operating lifespan	50,000 h
Electrolysis system lifespan	15 years
PV and wind system costs	
PV-initial investment expenses	$ 1296/kWp
PV-annual operational costs (relative to initial investment)	3.0%
Wind-initial investment expenses	$ 1080/kWp
Wind-annual operational costs (relative to initial investment)	3.8%

### Operational context and system constraints

This research focuses on large-scale hydrogen production using a 100 MWp renewable energy system in remote areas, including Egypt and other countries with similar conditions such as Jordan, UAE, Libya, Saudi Arabia, France, and Italy. These locations are characterized by limited or absent conventional grid infrastructure, making them ideal for off-grid hydrogen production. The system operates independently from the national grid, using only the renewable energy generated for electrolysis, with any excess electricity either curtailed or dissipated. Integrating grid electricity to lower production costs is not considered, as the analysis is focused on scenarios where extensive grid infrastructure is economically impractical.

Initially, hydrogen storage and transportation costs are excluded from the Levelized Cost of Hydrogen (LCOH) calculations due to their variability, which depends on transport distance, hydrogen carrier type (e.g., gaseous, liquid, ammonia, methanol), and transport mode (e.g., ship, pipeline, truck). These costs will be included in the Levelized Supply Cost of Hydrogen (LSCOH) for a more comprehensive cost assessment.

Water desalination energy requirements, essential in areas with limited water resources, are not included in the initial LCOH calculations. The impact of desalination on total energy consumption is minimal, estimated at around 0.1% of the electrolysis system’s energy demand. Investment costs for desalination range from $2.5 to $8.0/kW relative to electrolysis capacity, with fixed annual operational costs ranging from $0.20 to $0.80/kW. These costs are included in the overall installation and operating expenses, which are approximately $45.00/kW for the electrolysis system.

The renewable energy facility is assumed to use technology suited to high temperatures and dust conditions typical of these regions. While this study does not address potential grid extension and integration strategies, future research may explore hybrid systems combining off-grid production with intermittent grid support to enhance cost-effectiveness and reliability.

### Hydrogen production scenarios and regional evaluations

The selection of locations for hydrogen production was guided by several critical factors, including the diversity of renewable energy potentials and established hydrogen production strategies.

Egypt was chosen for its favorable renewable energy conditions. Within Egypt, Zafarana and Aswan were selected for their high energy yields in photovoltaic (PV) and wind energy, respectively. Zafarana was also analyzed for a combined PV and wind energy mix scenario.

Saudi Arabia was included due to its significant renewable energy potential. Al Khobar and Riyadh were selected for their strong PV and wind energy yields, respectively, while Jeddah was chosen for a combined energy mix scenario.

The UAE was represented for its substantial PV potential. Locations such as Al Ruwais, Dubai, and Abu Dhabi were selected based on their renewable energy output and strategic relevance.

Additionally, Jordan, Libya, and Italy were considered. Jordan and Libya were included for their emerging renewable energy potentials and regional strategic importance, while Italy was selected for its well-established renewable energy infrastructure.

Table 4 summarizes the selected hydrogen production sites, including annual energy yields for each renewable energy scenario, the Weighted Average Cost of Capital (WACC) values for 2021 and 2022, and the transport distances by pipeline and ship to Egypt. This comprehensive selection provides a broad spectrum of locations for assessing hydrogen production and transportation efficiencies across different regions

**Table 4:** Energy data for different locations.

Country	Location	RES-type	Annual energy yield [kWh/kWp/a]	WACC 2022E [%]	WACC 2021 [%]	Transport distance pipeline [km]	Transport distance ship [km]
Egypt	Zafarana	100 MWp PV	1907	8.23	4.49	0	0
		100 MWp Wind	3078	8.23	4.49	0	0
		Mix PV & Wind	2493	8.23	4.49	0	0
Tunisia	Sidi Daoud	100 MWp PV	1672	9.45	5.34	2500	2200
		100 MWp Wind	1898	9.45	5.34	2500	2200
		Mix PV & Wind	1785	9.45	5.34	2500	2200
UAE	Al Ruwais	100 MWp PV	1820	5.19	2.35	2400	3500
Jordan	Amman	100 MWp PV	1800	8.00	7.00	300	350
		100 MWp Wind	1500	8.00	7.00	300	350
		Mix PV & Wind	1650	8.00	7.00	300	350
Saudi Arabia	Riyadh	100 MWp PV	2200	7.00	6.00	1200	3000
		100 MWp Wind	1500	7.00	6.00	1200	3000
		Mix PV & Wind	1850	7.00	6.00	1200	3000
Libya	Tripoli	100 MWp PV	2100	11.00	10.00	500	900
		100 MWp Wind	1800	11.00	10.00	500	900
		Mix PV & Wind	1950	11.00	10.00	500	900
Italy	Rome	100 MWp PV	1500	4.00	3.00	1300	1500
		100 MWp Wind	2000	4.00	3.00	1300	1500
		Mix PV & Wind	1750	4.00	3.00	1300	1500
France	Paris	100 MWp PV	1400	5.00	4.00	2500	1500
		100 MWp Wind	1800	5.00	4.00	2500	1500
		Mix PV & Wind	1600	5.00	4.00	2500	1500

## Hydrogen levelized costs based on plant size and location

**Fig. 3:**
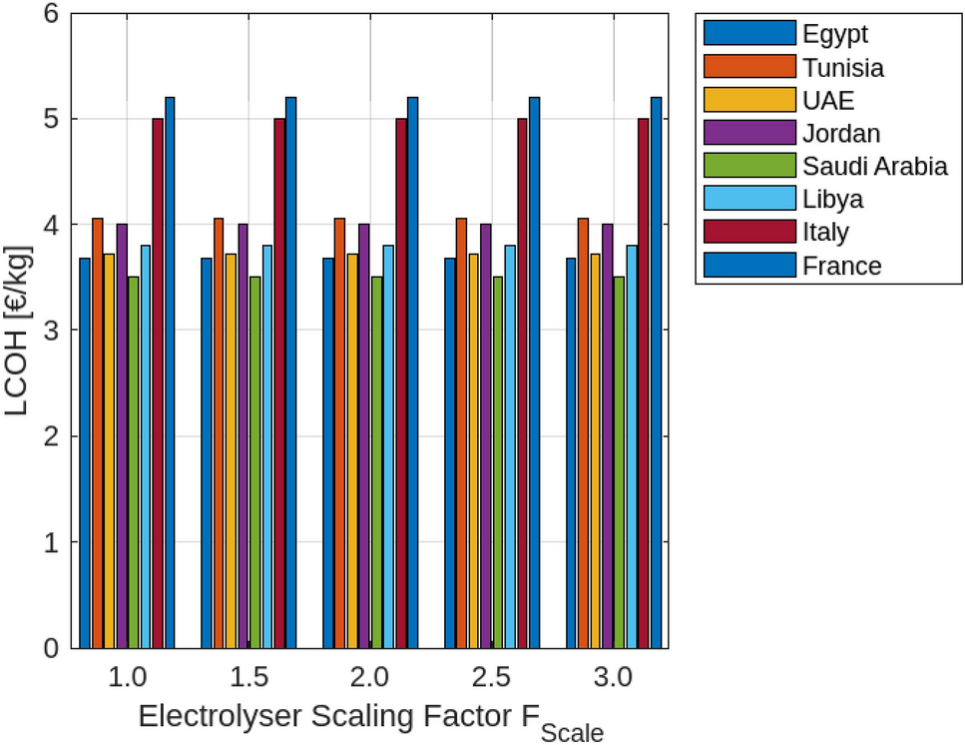
The effect of $F_{\text {Scale}}$ on different countries.

Designing a hydrogen production system for off-grid applications involves optimizing the balance between the generated energy and the operational hours of the electrolysis system. The effectiveness of this configuration depends on location-specific factors and the characteristics of available energy sources. As demonstrated in Fig. 3, a simulation assessed the most cost-effective setup for a 100 MWp renewable power capacity with electrolysis systems ranging from 10 to 100 MW. The study’s results can be extrapolated to larger renewable capacities using a scaling factor, $F_{\text {Scale}}$, defined as the ratio of renewable peak power ($R_{\text {RESP}}$) to installed electrolyser power ($R_{\text {ELY}}$):

5 $$\begin{aligned} F_{\text {Scale}} = \frac{R_{\text {RESP}}}{R_{\text {ELY}}} \end{aligned}$$

For electrolyser capacities below 10 MW, economies of scale play a crucial role in affecting efficiency and cost. As the scale of the electrolyser increases, the per-unit cost typically decreases due to more efficient use of resources and reduced operational costs. Incorporating energy storage systems can further enhance flexibility, managing intermittent renewable energy and reducing reliance on high-capacity electrolyzers. Economic considerations, including local energy costs and policy incentives, are vital for determining financial feasibility. Policy incentives and local energy prices can significantly influence the economic viability of hydrogen production. Table 5 provides a detailed breakdown of cost shares for cost-optimal off-grid electrolysis systems across various countries and renewable energy sources. The Levelized Cost of Hydrogen (LCOH) varies, with Egypt showing the lowest LCOH values at 3.68 ${\EUR \text{\euro } }$/kg for PV and 3.74 ${\EUR \text{\euro } }$/kg for wind, indicating a favorable economic environment for hydrogen production. The distribution of Capital Expenditure (CapEx) between electrolyser systems and renewable energy installations varies by country; Egypt and Tunisia allocate a larger portion to RES installation, while Libya and Italy exhibit a more balanced distribution. Operational Expenditure (OpEx) also shows variability, with Egypt managing lower OpEx shares compared to other regions. Additionally, mixed RES scenarios generally yield lower LCOH compared to single RES types, highlighting the cost benefits of combining multiple energy sources.The use of EUR for the current cost table and figres reflects the regional economic context of the European and nearby countries under analysis, providing more relevant financial insights compared to the USD used in the previous sections for a global perspective.

**Table 5:** Cost shares of the total expenses (TotEx) for the cost-optimal designed off-grid electrolysis systems of the scenarios under consideration.

Country	RES type	LCOH$_\text {IIR}$ 4%	CapEx share on TotEx			OpEx share on TotEx	
			Electrolysis	RES installation	Installation	Electrolysis	RES
Egypt	PV	3.68 ${\EUR \text{\euro } }$ /kg	25%	39%	12%	18%	6%
	Wind	3.74 ${\EUR \text{\euro } }$ /kg	20%	42%	10%	14%	15%
	Mix	3.38 ${\EUR \text{\euro } }$ /kg	19%	47%	9%	12%	13%
Tunisia	PV	4.06 ${\EUR \text{\euro } }$ /kg	25%	39%	12%	18%	6%
	Wind	5.85 ${\EUR \text{\euro } }$ /kg	17%	42%	9%	12%	13%
	Mix	4.43 ${\EUR \text{\euro } }$ /kg	18%	46%	9%	13%	16%
UAE	PV	3.72 ${\EUR \text{\euro } }$ /kg	25%	39%	12%	13%	6%
Jordan	PV	4.00 ${\EUR \text{\euro } }$ /kg	24%	38%	12%	17%	7%
	Wind	4.50 ${\EUR \text{\euro } }$ /kg	19%	41%	11%	14%	14%
	Mix	4.20 ${\EUR \text{\euro } }$ /kg	20%	45%	10%	13%	12%
Saudi Arabia	PV	3.50 ${\EUR \text{\euro } }$ /kg	23%	37%	12%	18%	8%
	Wind	4.70 ${\EUR \text{\euro } }$ /kg	18%	40%	11%	15%	16%
	Mix	4.00 ${\EUR \text{\euro } }$ /kg	21%	44%	10%	14%	13%
Libya	PV	3.80 ${\EUR \text{\euro } }$ /kg	26%	40%	12%	17%	7%
	Wind	4.90 ${\EUR \text{\euro } }$ /kg	20%	43%	11%	13%	15%
	Mix	4.30 ${\EUR \text{\euro } }$ /kg	22%	47%	10%	12%	13%
Italy	PV	5.00 ${\EUR \text{\euro } }$ /kg	21%	38%	12%	15%	9%
	Wind	5.50 ${\EUR \text{\euro } }$ /kg	16%	41%	11%	14%	14%
	Mix	5.20 ${\EUR \text{\euro } }$ /kg	18%	45%	10%	13%	12%
France	PV	5.20 ${\EUR \text{\euro } }$ /kg	22%	37%	12%	16%	8%
	Wind	5.70 ${\EUR \text{\euro } }$ /kg	17%	40%	11%	15%	13%
	Mix	5.40 ${\EUR \text{\euro } }$ /kg	19%	44%	10%	14%	11%

### Impact of electrolyser scaling on hydrogen production costs and energy utilization

The analysis of electrolyser scaling effects on hydrogen production costs and energy utilization provides valuable insights into optimizing renewable energy systems, particularly in diverse geographical contexts.

**Fig. 4:**
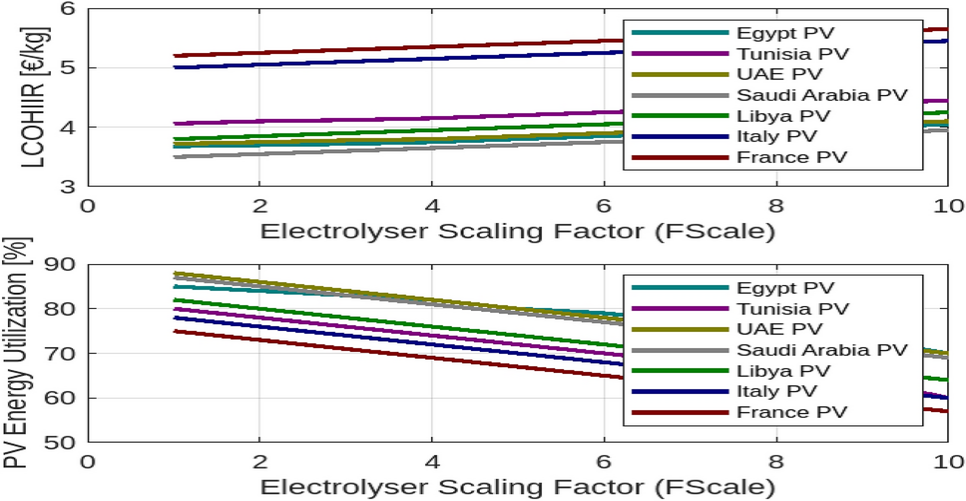
The effect of $F_{\text {Scale}}$ on different countries.

Figure 4 illustrates how varying the electrolyser scaling factor affects the Levelized Cost of Hydrogen (LCOH) and the utilization of photovoltaic (PV) and wind energy across different countries.

**Fig. 5:**
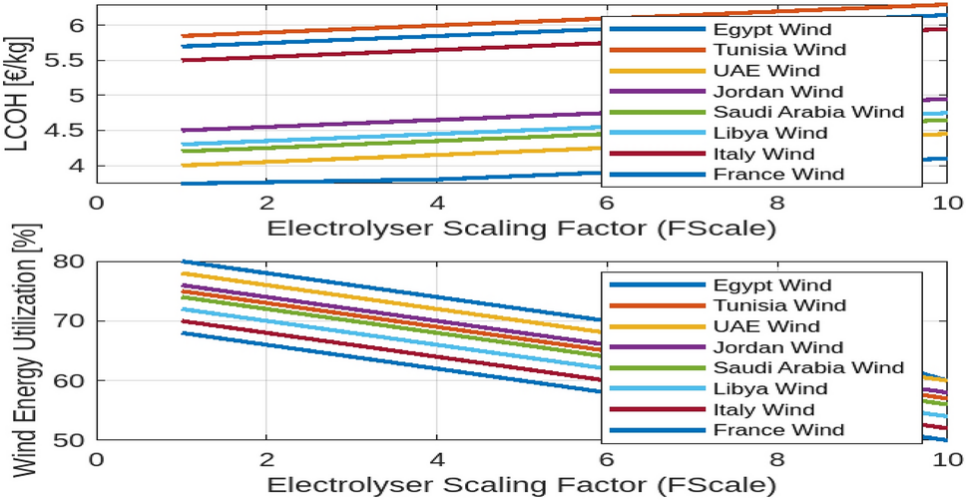
The effect of $F_{\text {Scale}}$ on different countries.

**Fig. 6:**
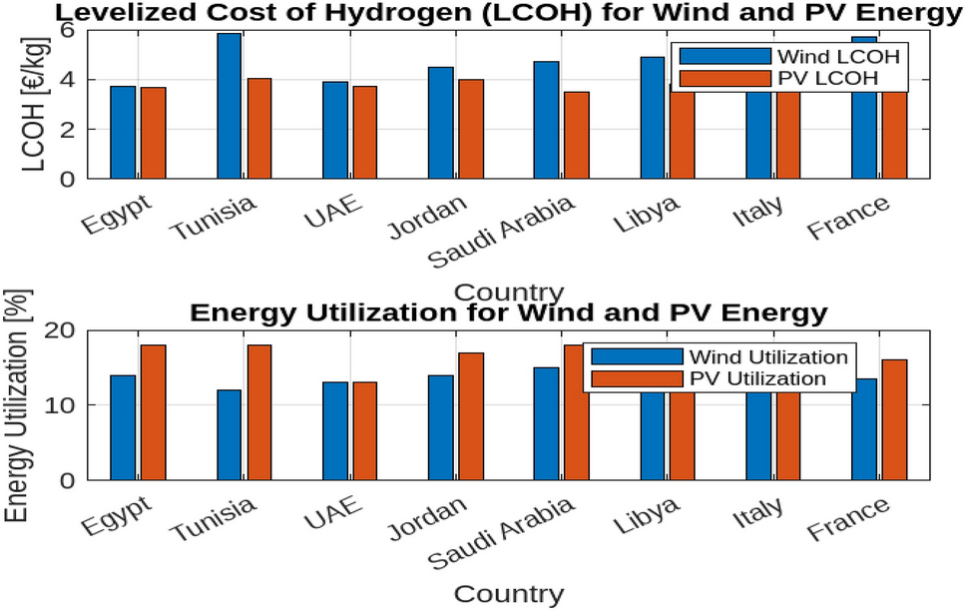
The effect of $F_{\text {Scale}}$ on different countries.

In the first subplot, the data reveal that LCOH generally increases with the scaling factor across all countries, though the rate of increase varies. For instance, Egypt experiences a more gradual rise in LCOH compared to Tunisia or France, indicating that scaling up the electrolyser has a less pronounced impact on costs in Egypt. Conversely, countries such as Saudi Arabia and Libya also exhibit an increasing trend in LCOH with scaling, but at different rates. This suggests that while increasing electrolyser capacity tends to elevate costs, the extent of this effect varies significantly depending on the country’s economic and resource context. The second subplot of Fig. 4 shows that the utilization of PV energy decreases as the scaling factor increases across all countries. This trend indicates that as the electrolyser’s capacity expands, the proportion of PV energy utilized declines. The reduction is more significant in countries with initially higher PV utilization, such as Egypt, whereas countries like Tunisia and France experience a more pronounced decline. This reflects the challenge of maintaining high efficiency in PV energy usage as system size grows. Fig. 5 examines the impact of electrolyser scaling on both LCOH and wind energy utilization across different locations. As $F_{\text {Scale}}$ increases, LCOH generally rises due to the higher capital costs associated with larger electrolyser systems. Concurrently, wind energy utilization tends to decrease, suggesting that larger systems may lead to less efficient use of wind resources. This implies that while scaling up electrolyser capacity can drive up hydrogen production costs, it may also result in suboptimal use of wind energy, highlighting the importance of careful system design to balance these factors. Figure 6 further illustrates the impact of the electrolyser scaling factor ($F_{\text {Scale}}$) on LCOH and the utilization of both wind and PV energy. As the scaling factor increases, LCOH rises due to higher capital expenditures, and energy utilization for both wind and PV decreases. This suggests that larger systems may not fully capitalize on available renewable energy resources. The Levelized Cost of Energy (LCOE) shows a similar trend, with higher scaling factors generally leading to increased costs per unit of energy produced. These findings underscore the need to balance electrolyser capacity and energy utilization to achieve economic efficiency in renewable energy systems.

Notably, Egypt has the lowest LCOH, at ${\EUR \text{\euro } }$3.68/kg for PV and ${\EUR \text{\euro } }$3.74/kg for wind, while Italy has the highest, at ${\EUR \text{\euro } }$5.00/kg for PV and ${\EUR \text{\euro } }$5.50/kg for wind. Egypt also demonstrates the highest energy utilization, with 85% for PV and 80% for wind, compared to lower rates in Tunisia and Libya. These figures highlight the trade-offs between scaling electrolyser capacity, hydrogen production costs, and energy utilization, emphasizing the importance of optimizing system design to balance cost and efficiency across different energy sources and locations.

### Impact of location, IIR, and WACC on Hydrogen production costs

To explore how economic factors affect off-grid electrolysis systems, we varied the Imputed Interest Rate (IIR) and the country-specific Weighted Average Cost of Capital (WACC). For the base scenario, we set an annual IIR at 3%

#### Off-grid electrolyzers powered by photovoltaic systems

**Fig. 7:**
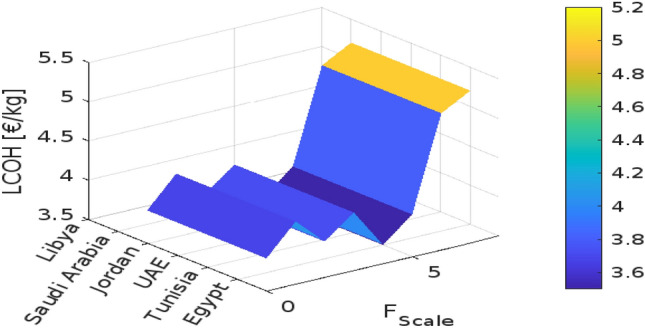
Impact of diffrent factors on LCOH for Off-Grid PV electrolysis systems.

**Fig. 8:**
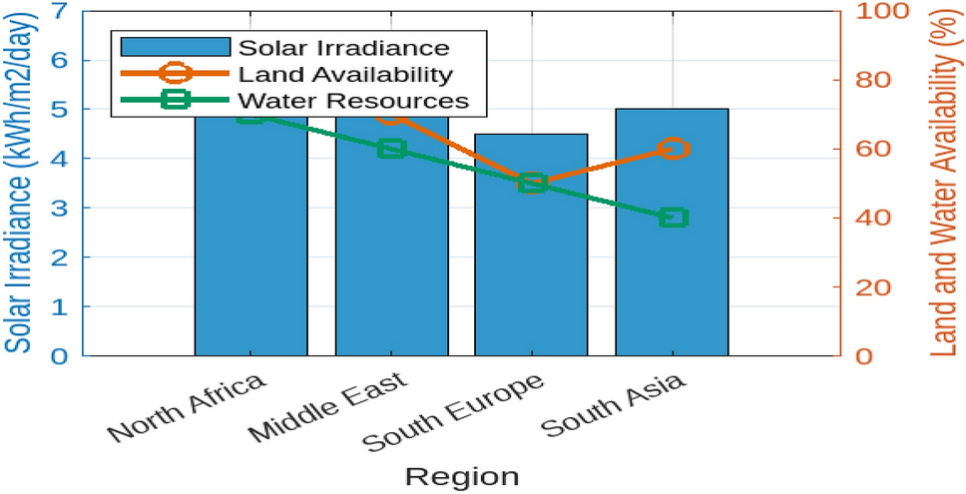
Geographical suitability for PV-supplied off-grid electrolysers.

**Fig. 9:**
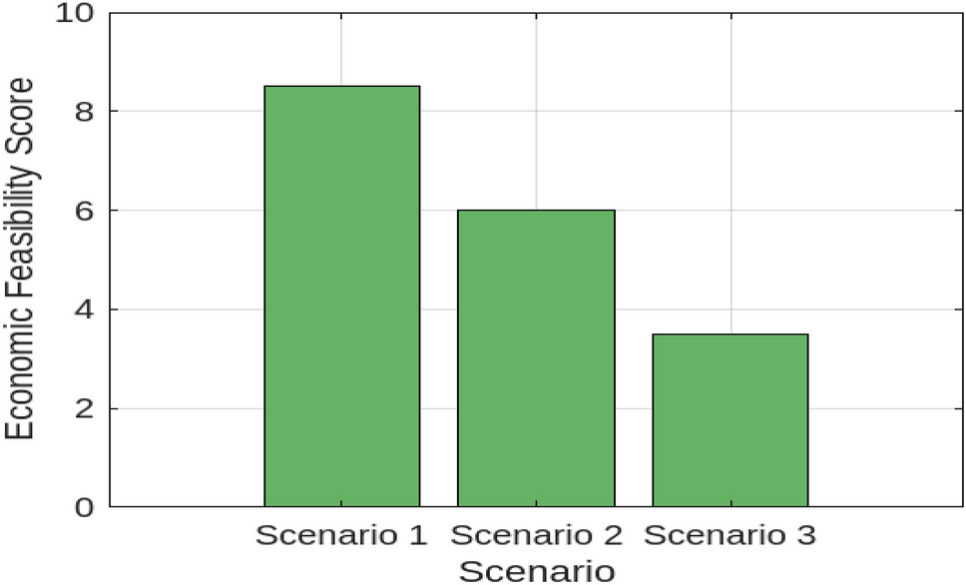
Economic feasibility of PV-supplied off-grid electrolysers.

To understand how hydrogen production costs are influenced by various factors, we first examined the Levelized Cost of Hydrogen (LCOH) across different locations and operational conditions. Figure 7 illustrates the resulting LCOH for 10–100 MW electrolysis systems with a 100 MWp PV off-grid supply. This figure provides insight into how the LCOH varies with different operational conditions and locations, revealing the relationship between system size, location, and hydrogen production costs. Next, to assess the geographical suitability for PV-supplied off-grid electrolysers, we evaluated solar irradiance, land availability, and water resources across various regions. Figure 8 offers a comprehensive view of these factors. The bar chart on the left demonstrates that the Middle East has the highest solar irradiance at 6.0 kWh/m$^2$/day, which is superior to North Africa and South Europe. This advantage positions the Middle East favorably for solar energy generation. Conversely, South Europe, with the lowest solar irradiance at 4.5 kWh/m$^2$/day, may face challenges in achieving similar energy production levels. The accompanying plot on the right details land availability and water resources, showing North Africa’s high land availability at 80%, making it highly suitable for large-scale PV installations. In contrast, South Asia’s limited water resources at 40% could impact the feasibility of PV systems that require substantial water for maintenance. To evaluate the economic feasibility of PV-supplied off-grid electrolysers under different technological scenarios, we analyzed three distinct configurations with varying levels of technological advancement and cost implications. Figure 9 explores these scenarios:

- **Scenario 1** features the most technologically advanced configuration, integrating the latest developments in PV panels and electrolyser technologies. This setup includes high-efficiency PV panels, state-of-the-art electrolyser designs, and optimized storage solutions. The advanced technology aims to minimize both capital and operational costs, resulting in a superior economic feasibility score of 8.5 out of 10.
- **Scenario 2** represents a more conventional approach, utilizing standard PV panels and electrolyser technologies along with average storage solutions. This setup achieves a moderate economic feasibility score of 6.0, reflecting a balanced solution with reasonable performance and costs.
- **Scenario 3** illustrates the least favorable configuration, incorporating outdated technology and higher costs. This scenario uses older-generation PV panels and electrolyser designs, resulting in increased capital and operational costs, and received the lowest score of 3.5.

**Fig. 10:**
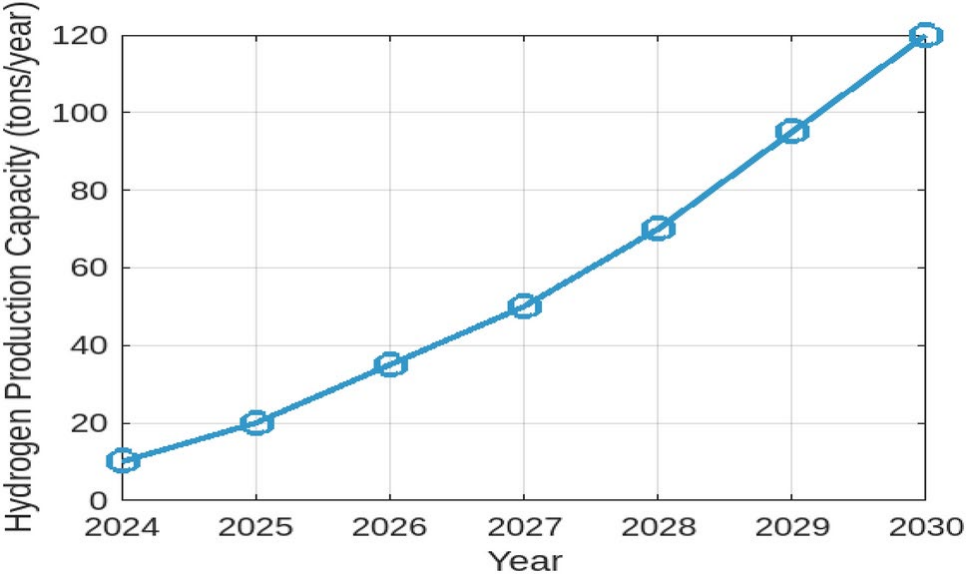
Projected hydrogen production capacity from PV-supplied off-grid electrolysers.

**Fig. 11:**
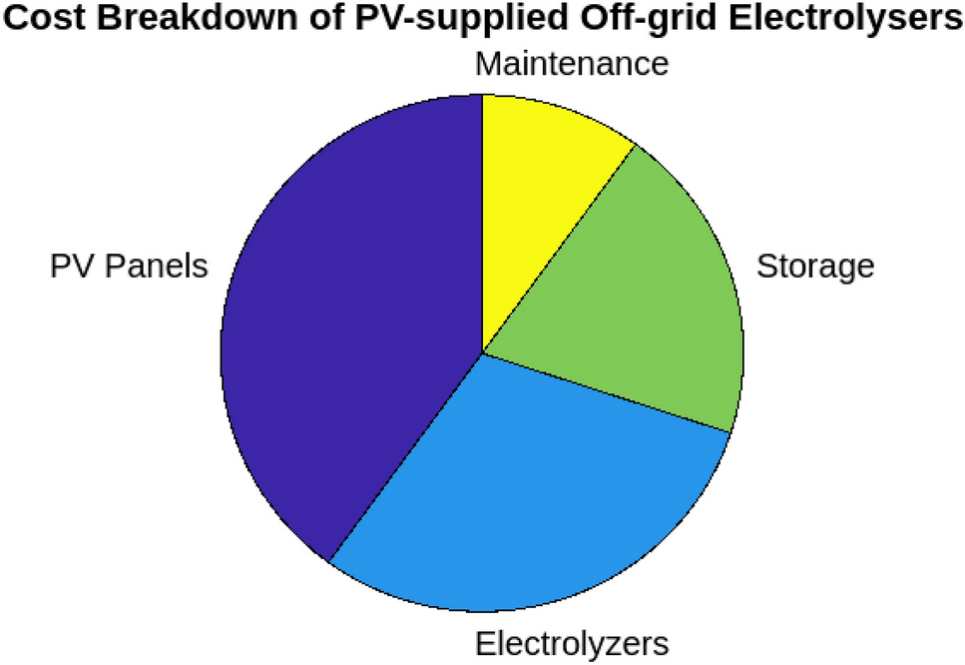
Cost breakdown of PV-supplied off-grid electrolysers.

**Fig. 12:**
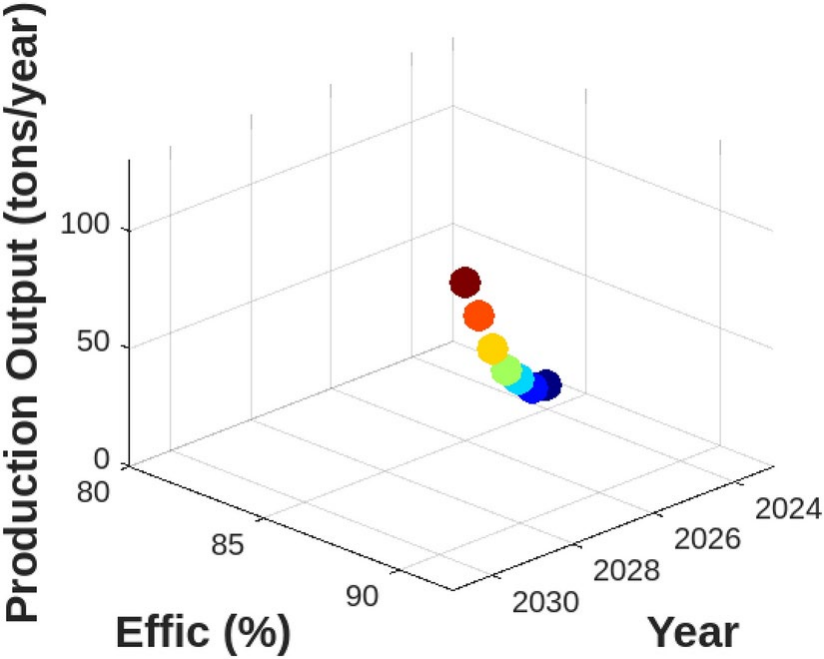
Performance over time for PV-supplied off-grid electrolysers.

These scenarios highlight the impact of technological advancements and cost considerations on economic feasibility, with Scenario 1 offering the most promising financial outlook. To project future hydrogen production potential, we analyzed the expected growth in production capacity from 2024 to 2030. Figure 10 shows a significant increase from 10 to 120 tons/year. This growth underscores the potential for scaling up hydrogen production in Egypt, driven by advancements in PV technology and electrolyser performance. Understanding the financial aspects of PV-supplied off-grid electrolysis systems is crucial for effective financial planning. Figure 11 illustrates the cost breakdown for these systems. The pie chart reveals that PV panels account for the largest share of the total cost at 40%, followed by electrolyzers at 30%, storage solutions at 20%, and maintenance at 10%. This distribution highlights the financial impact of key components and helps in optimizing project expenditures. Finally, to evaluate how technological improvements affect system performance over time, Fig. 12 shows a dual-axis line plot of PV-supplied off-grid electrolysers. The graph indicates improvements in efficiency from 85% in 2024 to 91% in 2030, and in capacity utilization from 70 to 85%, alongside a significant increase in production output from 10 to 120 tons/year. These improvements reflect the positive impact of technological progress on system performance and hydrogen production.

**Table 6:** Dependence of LCOH on changes in IIR, WACC, and other factors.

Energy supply	Impact category	Mean change in LCOH [%]	Location dependency	Capacity factor	Operational efficiency	Capital cost
PV energy supply	IIR sensitivity	8.6%	$$\pm 0.5$$ %pt	5%	90%	$1.3M
	WACC sensitivity	7.9%	$$\pm 0.4$$ %pt	5%	90%	$1.3M
Wind energy supply	IIR sensitivity	8.1%	$$\pm 0.3$$ %pt	6%	85%	$1.6M
	WACC sensitivity	7.3%	$$\pm 0.3$$ %pt	6%	85%	$1.6M
Mixed energy supply	IIR Sensitivity	8.2%	$$\pm 0.3$$ %pt	5.5%	87%	$1.5M
	WACC sensitivity	7.3%	$$\pm 0.3$$ %pt	5.5%	87%	$1.5M

Table 6 summarize the sensitivity of the Levelized Cost of Hydrogen (LCOH) to various financial and operational factors for PV, Wind, and Mixed energy supplies used in off-grid electrolysis systems. PV energy systems show a mean LCOH change of 8.6% due to Interest Rate (IIR) fluctuations and 7.9% due to the Weighted Average Cost of Capital (WACC), reflecting their sensitivity to financial changes. With a capital cost of $1.3 million, PV systems exhibit consistent performance with a 5% capacity factor and 90% operational efficiency. Wind energy systems, with a higher capital cost of $1.6 million, show slightly less sensitivity to financial changes, with a mean LCOH change of 8.1% for IIR and 7.3% for WACC. Wind systems have a higher capacity factor of 6% but lower operational efficiency at 85%. The Mixed energy supply approach, balancing the characteristics of both PV and wind, has a capital cost of $1.5 million and shows mean LCOH changes of 8.2% and 7.3% for IIR and WACC, respectively. This analysis underscores the influence of financial metrics on hydrogen production costs and highlights the trade-offs between different energy supply options.

#### Off-grid electrolyzers powered by wind systems

**Fig. 13:**
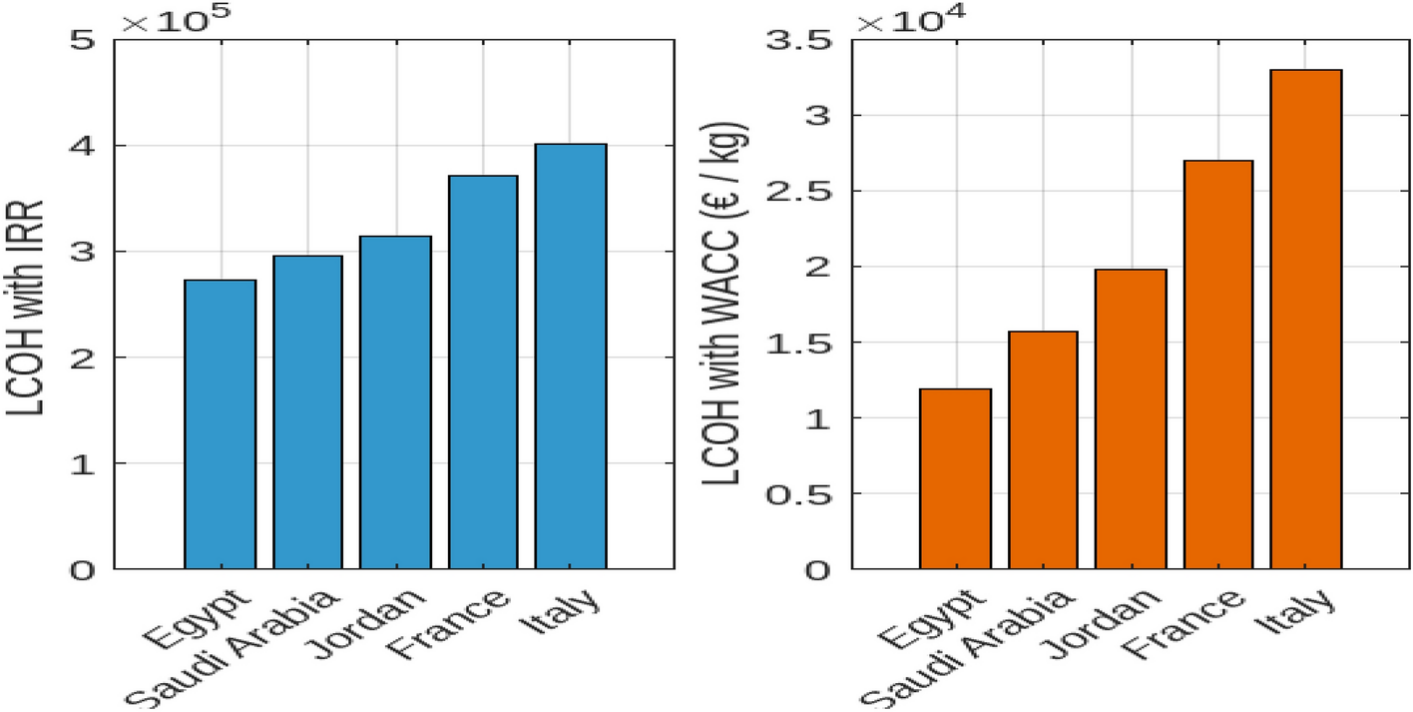
Cost analysis of hydrogen production using off-grid wind electrolysis. (**a**) Levelized cost of hydrogen with internal rate of return (LCOHIIR) across different countries. (**b**) Levelized cost of hydrogen with weighted average cost of capital (LCOHWACC) across different countries.

In this section, we examine the hydrogen production costs and capacities for off-grid wind electrolysis systems across various countries. Figure 13 presents the Levelized Cost of Hydrogen (LCOH) with Internal Rate of Return (LCOHIIR) and Weighted Average Cost of Capital (LCOHWACC) to assess cost variations based on Full Load Hours (FLH) and financial metrics.

**Fig. 14:**
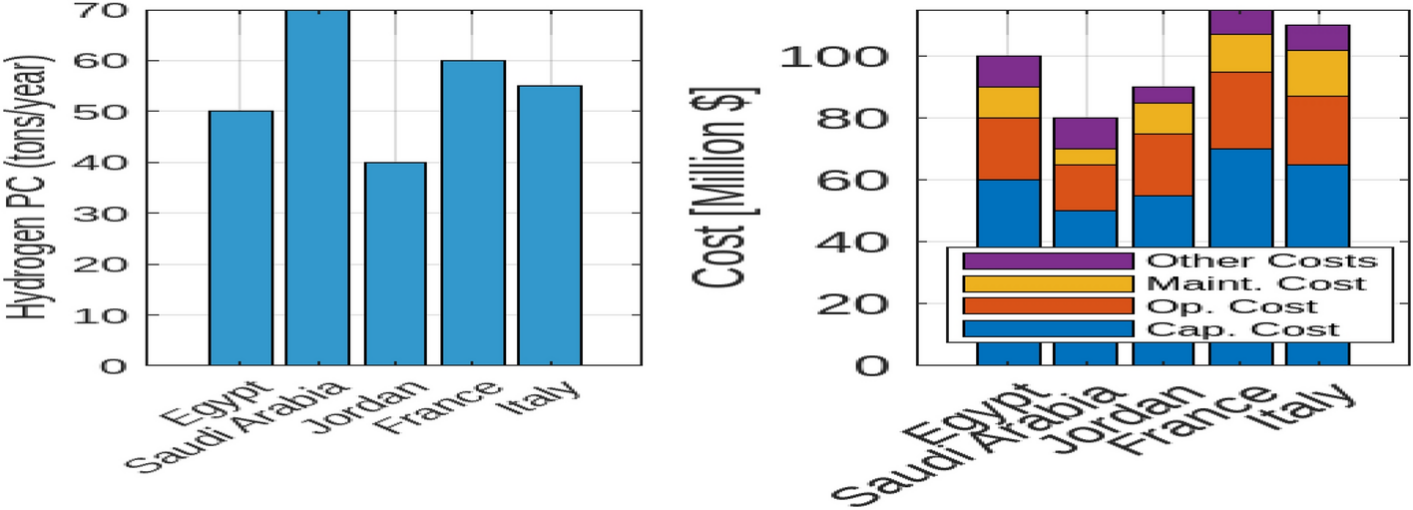
Hydrogen production capacity and financial breakdown for wind electrolysis systems. (**a**) Hydrogen production capacity (tons/year) across different countries. (**b**) Detailed cost breakdown of hydrogen production systems by component across different countries.

Subplot 1a of Fig. 13 illustrates LCOHIIR values for each country. Egypt, operating at 3670 FLH, achieves the lowest LCOHIIR of 3.7 ${\EUR \text{\euro } }$/kg, demonstrating the efficiency of its wind resources. Saudi Arabia and France show LCOHIIR values of 5.4 ${\EUR \text{\euro } }$/kg, with France operating at 2700 FLH and Saudi Arabia at 3400 FLH. Jordan’s LCOHIIR of 4.4 ${\EUR \text{\euro } }$/kg at 3200 FLH and Italy’s higher LCOHIIR of 5.6 ${\EUR \text{\euro } }$/kg at 2500 FLH further highlight the impact of FLH on production costs. This analysis underscores the importance of optimizing FLH to manage LCOH effectively. the other subplot continues the analysis with LCOHWACC, revealing the influence of Weighted Average Cost of Capital on hydrogen production costs. Despite Egypt’s lower LCOHIIR, its LCOHWACC shows competitive values compared to Saudi Arabia and France. This indicates that while Egypt benefits from lower LCOHIIR, the overall hydrogen production costs, when factoring in WACC, are more competitive in Egypt and Saudi Arabia relative to France. Balancing FLH and WACC is crucial for cost optimization across different regions. Figure 14 delves into hydrogen production capacity and cost breakdown. Subplot 2a presents hydrogen production capacities, with Egypt leading at 50 tons/year, Saudi Arabia at 70 tons/year, and France at 60 tons/year. Jordan and Italy exhibit capacities of 40 and 55 tons/year, respectively, while Libya matches Italy’s capacity. These differences reflect the varying efficiencies and scalability of wind energy systems based on regional conditions. Subplot 2b provides a detailed cost breakdown, highlighting capital, operational, maintenance, and other associated costs. The stacked bar chart shows capital costs as the largest expense, varying from 50 million in Saudi Arabia to 70 million in France. Operational costs are highest in France at 25 million and lowest in Saudi Arabia at 15 million. Maintenance costs are notably higher in France and Italy compared to other countries. This detailed financial analysis emphasizes the importance of considering both capital and operational expenditures when evaluating the feasibility of wind-based hydrogen production systems. In conclusion, this comprehensive analysis highlights Egypt and Saudi Arabia as more cost-effective locations for hydrogen production, driven by lower LCOHIIR and competitive LCOHWACC values.

#### Solar and Wind energy-fueled off-grid electrolysis systems

**Fig. 15:**
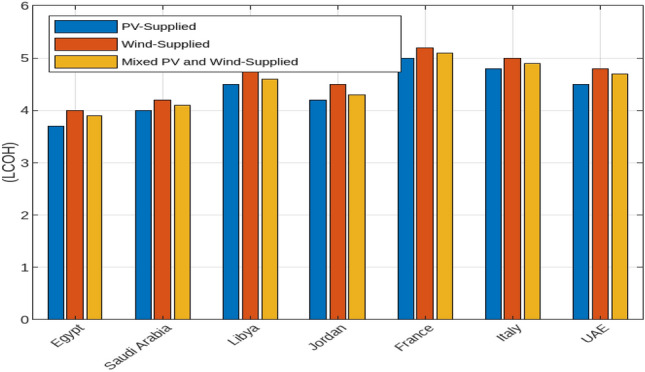
Comparative analysis of the Levelized Cost of Hydrogen (LCOH) for electrolysers powered by PV, wind, and a mix of both energy sources across various countries.

**Fig. 16:**
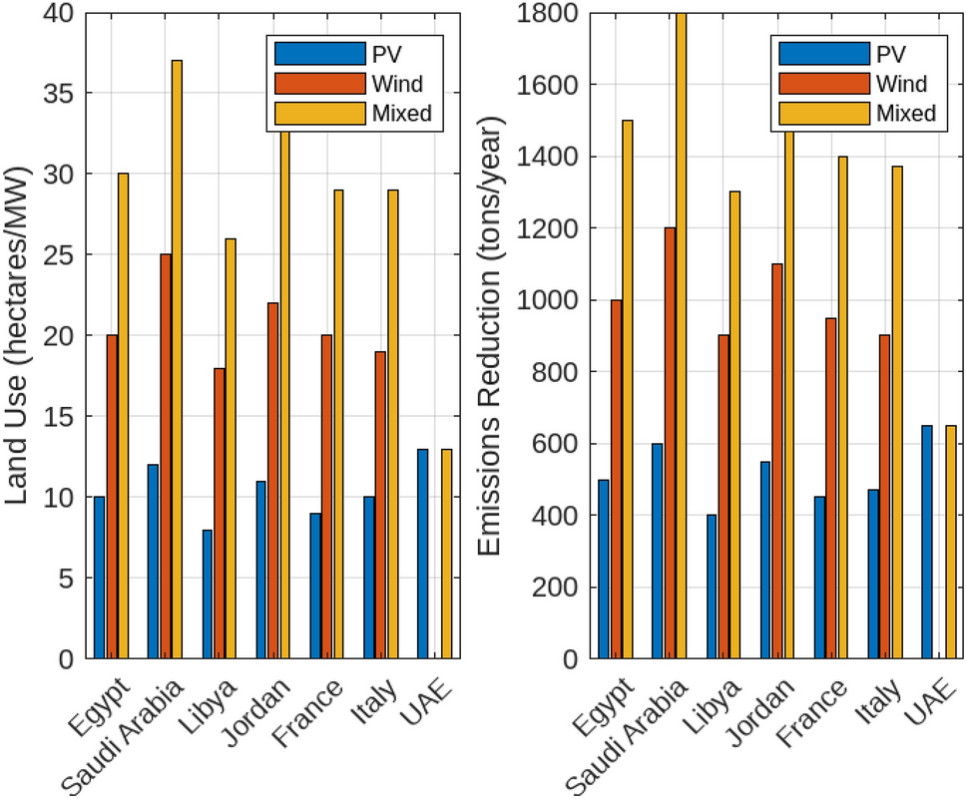
Environmental impact of PV and wind systems, as well as a combined PV and wind approach, in terms of land use and emissions reduction.

**Fig. 17:**
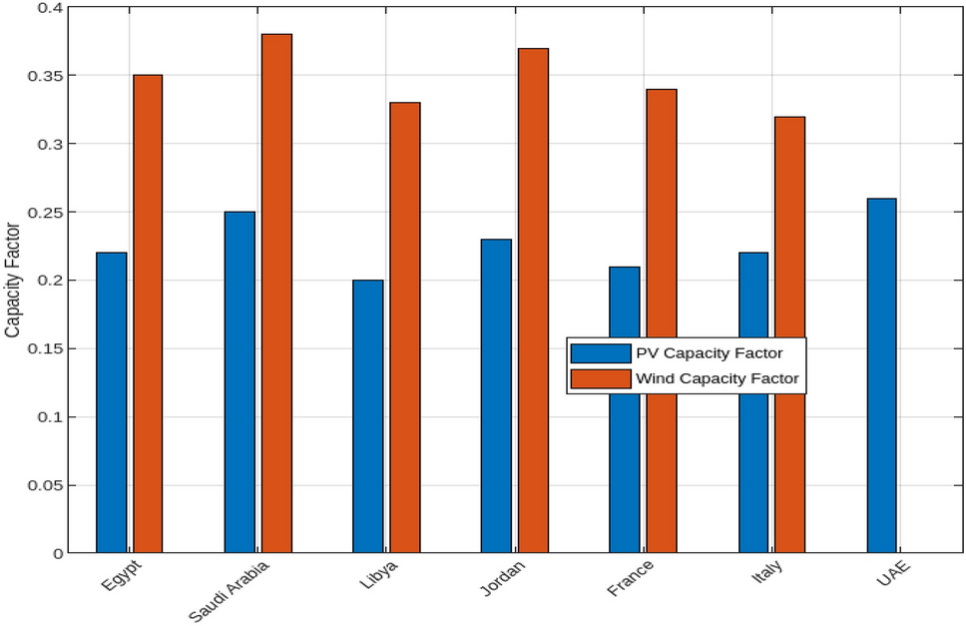
Capacity factors for PV and wind energy systems across various countries.

The economic and environmental impacts of photovoltaic (PV) and wind energy systems, including a combination of both, across various countries are discussed. In particular, the Levelized Cost of Hydrogen (LCOH), environmental impact in terms of land use and emissions reduction, and the capacity factors for PV and wind energy systems. Figure 15 presents a comparative analysis of the LCOH for electrolysers powered by PV, wind, and a mix of both energy sources. In Egypt, the LCOH is the lowest among the examined countries, with values of $3.7/kg for PV-supplied electrolysers, $4.0/kg for wind-supplied electrolysers, and $3.9/kg for those using both PV and wind energy. This highlights Egypt’s advantageous renewable energy conditions, making it a cost-effective location for hydrogen production. Conversely, Saudi Arabia has slightly higher LCOH values, with $4.0/kg for PV, $4.2/kg for wind, and $4.1/kg for the mixed approach. This trend indicates that while combining PV and wind reduces costs compared to wind alone, it does not achieve the cost-efficiency of PV alone. Libya and Jordan display similar patterns, where the mixed approach offers lower costs than wind alone but remains higher than PV alone. France and Italy show the highest LCOH values, with France at $5.0/kg for PV, $5.2/kg for wind, and $5.1/kg for mixed systems, and Italy at $4.8/kg for PV, $5.0/kg for wind, and $4.9/kg for mixed. The UAE, relying mainly on PV energy, achieves competitive LCOH values of $4.5/kg for PV, $4.8/kg for wind, and $4.7/kg for mixed systems, illustrating the cost benefits of integrating wind with PV. Figure 16 illustrates the environmental impact of PV and wind systems, as well as a combined PV and wind approach, in terms of land use and emissions reduction. Wind energy systems generally require more land per MW than PV systems, with Egypt and Saudi Arabia using about 10 and 12 hectares/MW for PV installations compared to approximately 20 hectares/MW for wind installations. The mixed systems show a higher aggregate land use due to the combined land requirements. Regarding emissions reduction, wind energy contributes significantly more to CO$$_2$$ reductions compared to PV, with reductions of around 1000 and 1200 tons/year in Egypt and Saudi Arabia, respectively. Although PV systems also contribute to emissions reduction, the impact is smaller. The mixed systems benefit from both sources, demonstrating higher overall emissions reductions, reflecting an enhanced environmental advantage. This underscores the trade-offs between land use and environmental impact in deploying PV, wind, or mixed energy systems. Figure 17 highlights the capacity factors for PV and wind energy systems across various countries. Saudi Arabia and Egypt achieve the highest capacity factors for PV systems, approximately 0.25 and 0.22 respectively, indicating their favorable solar conditions. In contrast, France and Italy exhibit lower capacity factors of around 0.21 and 0.22, possibly due to less optimal solar conditions or inefficiencies. For wind energy, Saudi Arabia and Egypt also show higher capacity factors of approximately 0.38 and 0.35, reflecting favorable wind conditions. The capacity factor of the mixed systems will result in higher capacity factors, reflecting the optimal utilization of both energy sources.

#### Hydrogen cost analysis in context of energy yield

**Fig. 18:**
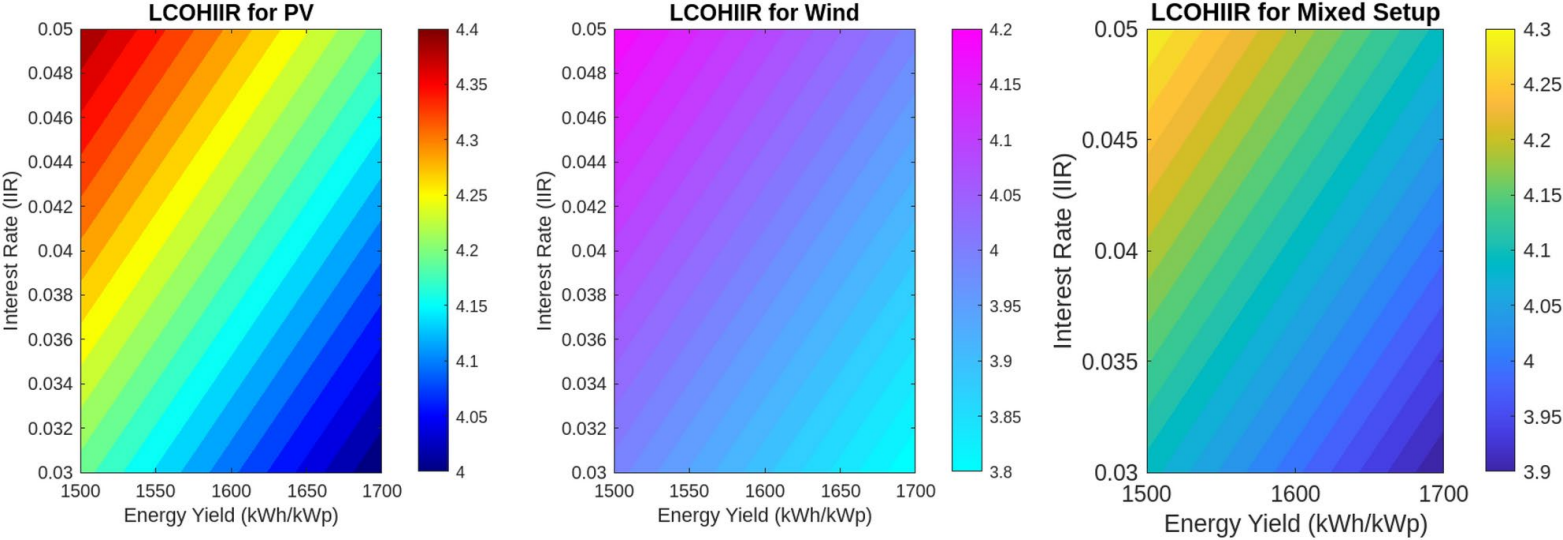
Impact of interest rates on levelized cost of hydrogen (LCOH) for different energy systems.

**Fig. 19:**
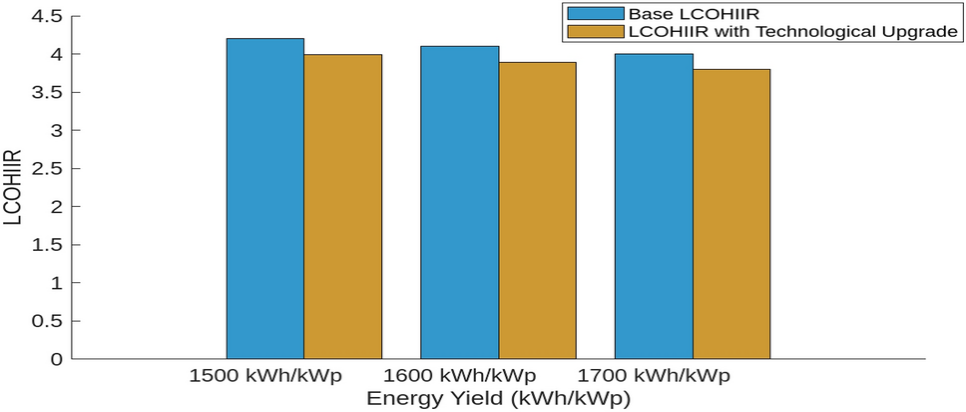
Effect of technological upgrades on levelized cost of hydrogen (LCOH).

**Fig. 20:**
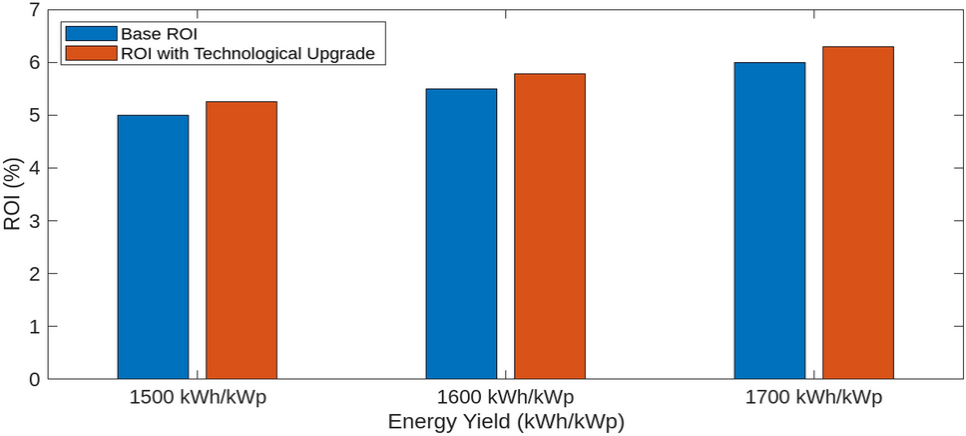
Return on investment (ROI) before and after technological upgrades.

**Fig. 21:**
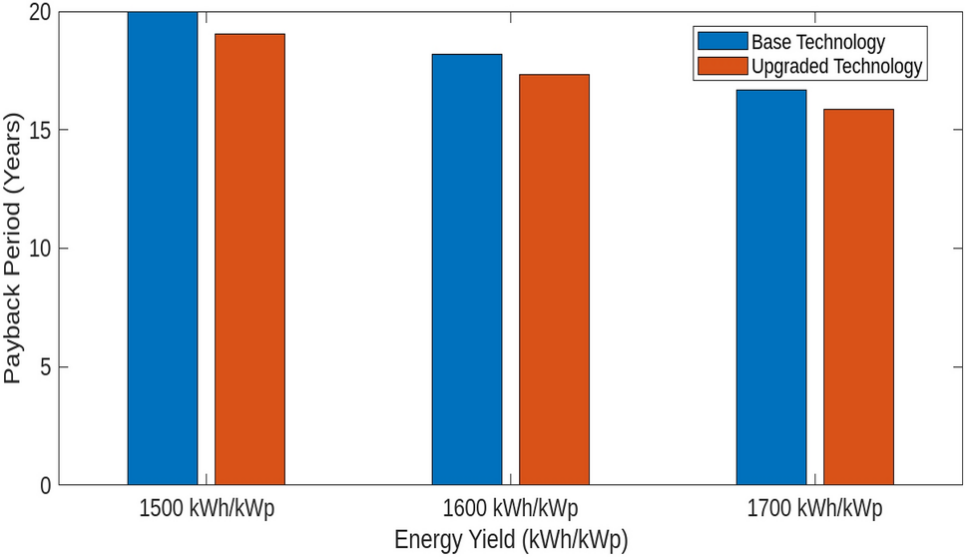
Payback period before and after technological upgrades.

**Fig. 22:**
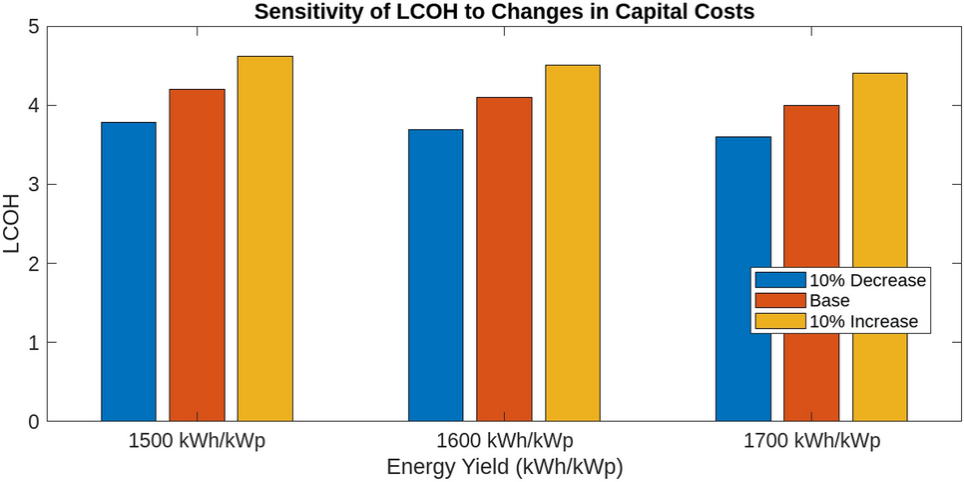
Impact of capital costs on levelized cost of hydrogen (LCOH).

This section investigates how variations in interest rates, technological upgrades, capital costs, and energy yields impact the Levelized Cost of Hydrogen (LCOH), Return on Investment (ROI), and payback periods for different energy systems. Using MATLAB simulations, we assess the effects of these factors on economic metrics To evaluate the impact of technological upgrades, we first establish a baseline using current technology and then simulate a 5% reduction in LCOH due to technological improvements. These upgrades include enhanced electrolyser efficiency, improved integration of renewable energy sources, and advanced materials and design. The upgraded scenario was tested in MATLAB, showing how these advancements reduce LCOH and improve financial metrics. Figure 18 illustrates the impact of varying Interest Rates (IIR)-3%, 4%, and 5%-on the LCOH for different energy systems. For PV systems, a 3% IIR results in an LCOH ranging from $4.0 to $4.2 per kilogram, with an average of $4.1. As the IIR increases to 4% and 5%, the LCOH rises to $4.1–$4.3 and $4.2–$4.4, respectively. This represents a 2.4–4.8% increase compared to the 3% IIR scenario. In contrast, wind systems exhibit greater sensitivity, with LCOH increasing from $3.8–$4.0 at 3% IIR to $3.9–$4.1 and $4.0–$4.2 at 4% and 5% IIR, respectively, reflecting increases of 2.6% and 5.3%. The mixed energy setup shows intermediate results, with LCOH increasing from $3.9–$4.1 at 3% IIR to $4.0–$4.2 and $4.1–$4.3 at higher rates, demonstrating increases of 2.5% and 5.0%. This analysis underscores the varying financial impacts of interest rates on different energy technologies. The effect of technological upgrades on LCOH is further examined in Fig. 19. The upgrades, simulated as a 5% reduction in LCOH, significantly enhance cost efficiency. This improvement is particularly pronounced at higher energy yields, highlighting the effectiveness of advancements such as enhanced electrolyser efficiency, improved renewable energy integration, and advanced materials and design. These upgrades contribute to reduced hydrogen production costs and improved economic viability. Figure 20 compares ROI before and after technological upgrades. The bar chart indicates a consistent improvement in ROI across all energy yield scenarios. For instance, the ROI for a 1500 kWh/kWp energy yield increases from 5.00 to 5.25%, reflecting a 5% enhancement due to the upgrades. This trend is evident for higher energy yields as well, demonstrating that technological upgrades enhance financial returns and make investments more attractive and profitable. Figure 21 illustrates the effect of technological upgrades on the payback period. The base technology shows longer payback periods, ranging from approximately 20–22 years. With technological upgrades, these periods decrease, with the payback period shortening from 20 years to about 19 years at an energy yield of 1500 kWh/kWp. This reduction highlights the improved financial efficiency and attractiveness of projects due to technological advancements. To further analyze the influence of capital costs on LCOH, Fig. 22 depicts the impact of a 10% reduction and increase in capital costs. A decrease in capital costs leads to a reduction in LCOH from $5.00, $5.50, and $6.00 to approximately $4.50, $4.95, and $5.40, respectively. Conversely, an increase in capital costs raises LCOH to about $5.50, $6.05, and $6.60. This direct correlation emphasizes the importance of managing capital costs to optimize hydrogen production economics and highlights the significant financial implications of capital cost fluctuations.

## Analysis of transport and infrastructure costs for hydrogen carriers

**Fig. 23:**
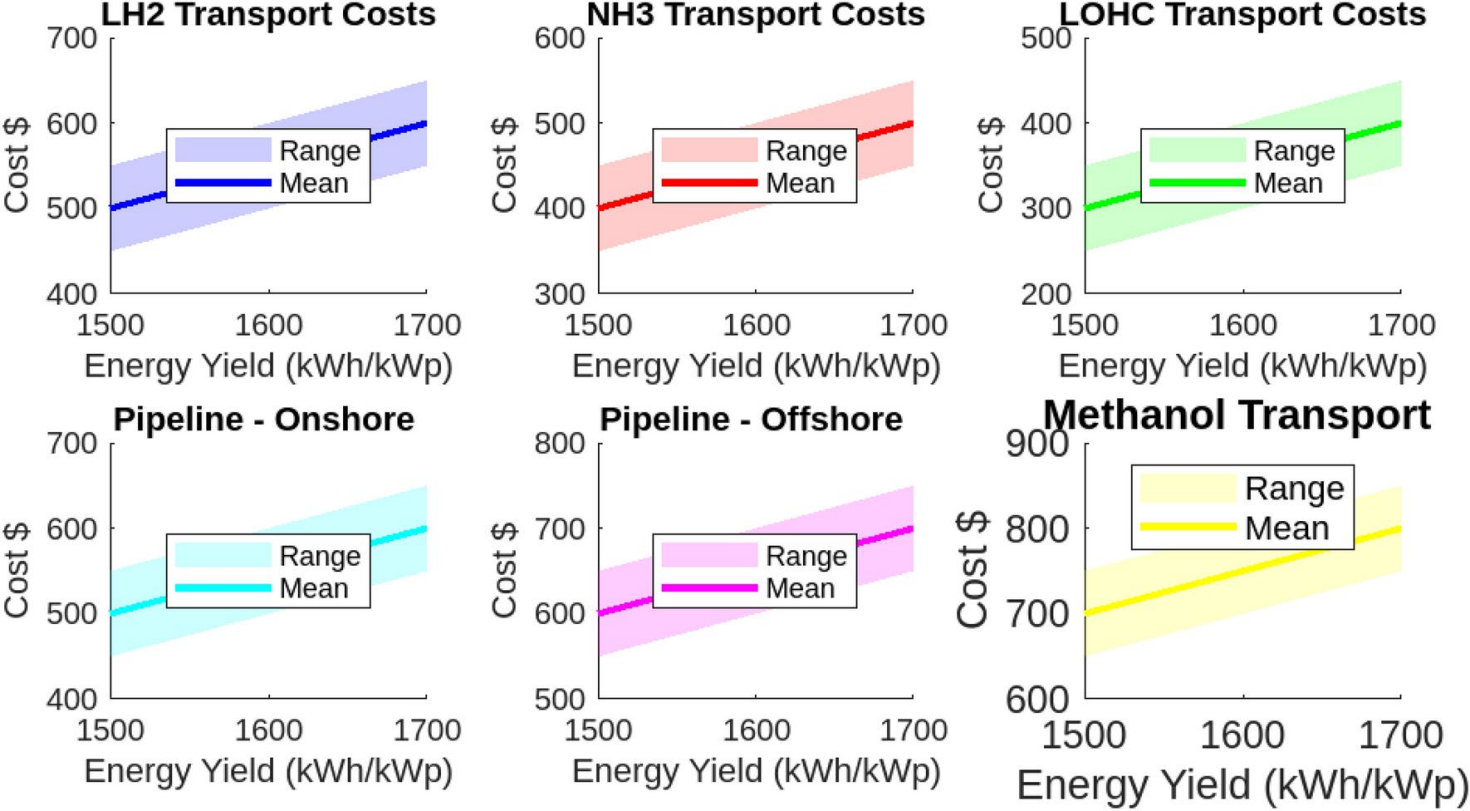
Hydrogen transport cost range.

**Fig. 24:**
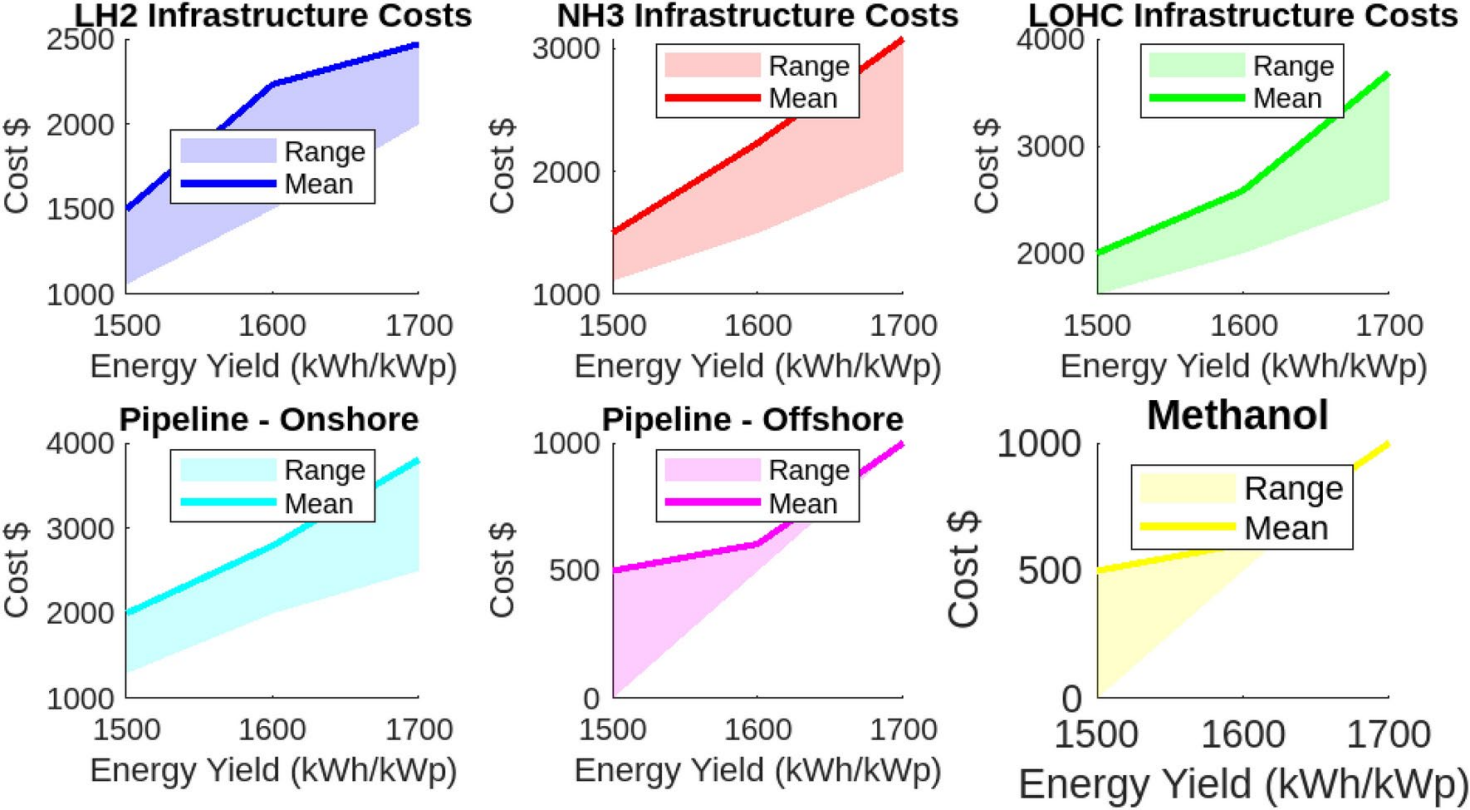
Hydrogen infrastructure cost range.

The analysis of transport and infrastructure costs for various hydrogen carriers reveals significant variability based on carrier type and transport distance. This section provides a detailed examination of these costs. Liquid Hydrogen (LH2) exhibits transport costs ranging from $0.084 to $0.345 USD/kg per 100 km. These costs increase notably with longer distances, such as those from France (3000 km). In contrast, Ammonia (NH3) presents a more economical alternative, with transport costs ranging from $0.042 to $0.1725 USD/kg. The Liquid Organic Hydrogen Carrier (LOHC) offers the lowest transport costs, ranging from $0.025 to $0.1035 USD/kg. Onshore pipeline transport costs vary from $0.215 to $0.885 USD/kg, while offshore pipelines are the most expensive, reaching up to $1.105 USD/kg. Methanol is noted for its cost-effectiveness, with transport costs ranging from $0.015 to $0.063 USD/kg. Infrastructure costs also show considerable variability. LOHC infrastructure is the most expensive, ranging from $1.617 to $3.685 USD/kg. In comparison, infrastructure costs for LH2 and NH3 are more moderate, with LH2 ranging from $1.06 to $2.47 USD/kg and NH3 from $1.111 to $3.080 USD/kg. Onshore pipeline infrastructure costs are significant, ranging from $1.298 to $3.806 USD/kg. Offshore pipeline infrastructure and methanol infrastructure are relatively less costly, with methanol being the least expensive at $0.0 to $0.605 USD/kg. For consistency in international comparisons and financial assessments, all calculations are presented in USD. These findings underscore the critical importance of considering both transport and infrastructure costs in hydrogen logistics planning. All analyses are illustrated in Figs. 23 and 24.

### Case study: Egypt

**Fig. 25:**
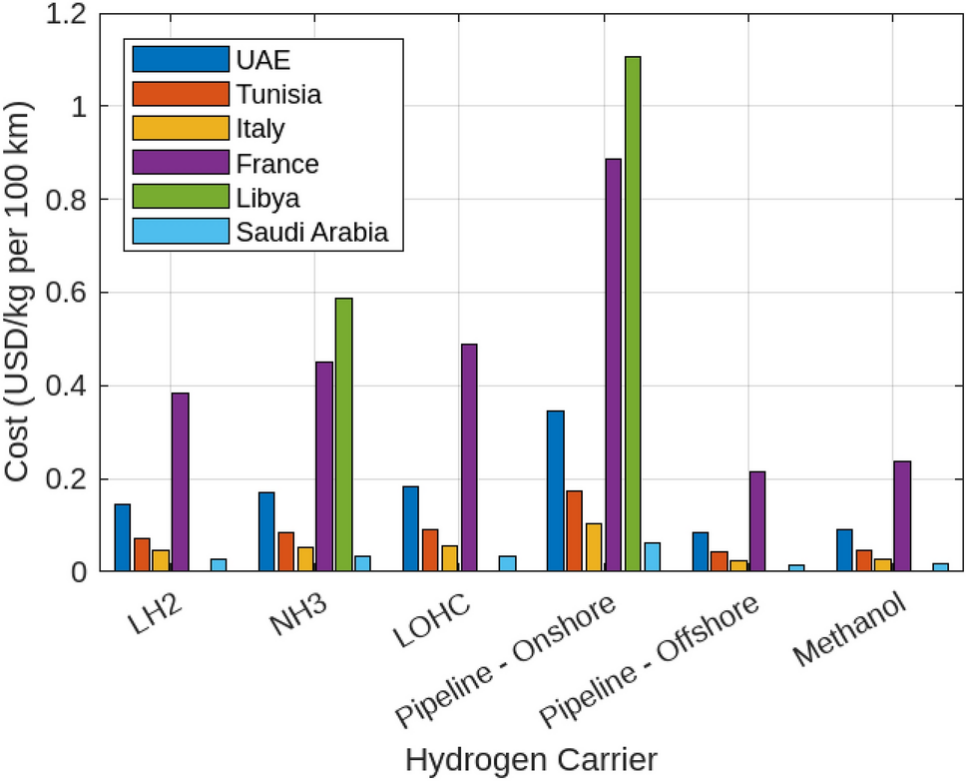
Comparative analysis of hydrogen transport costs by carrier and distance.

**Fig. 26:**
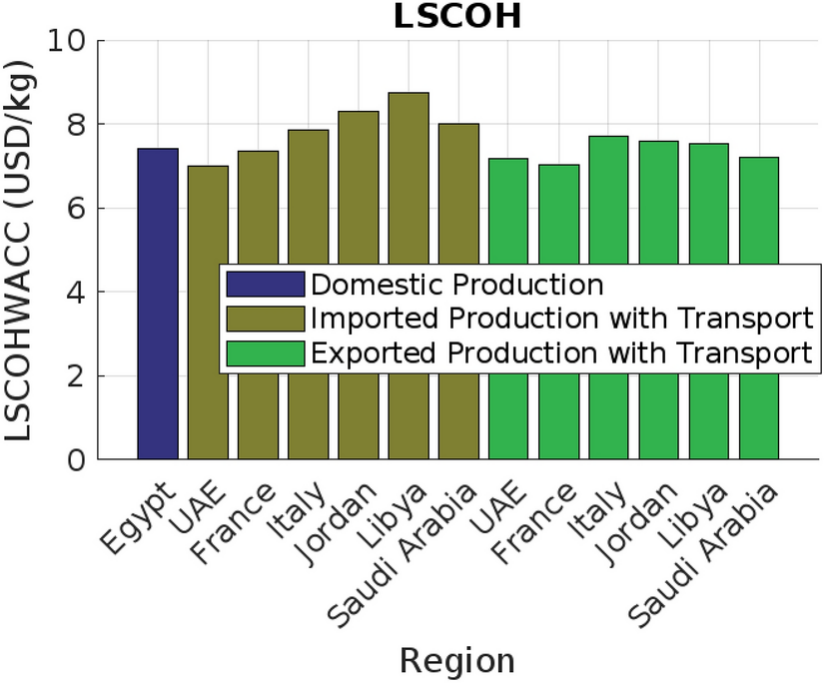
Levelized supply costs and transportation costs for hydrogen utilization in Egypt.

Focusing on Egypt, the country’s strategic position enhances its role as both a hydrogen importer and exporter. For transport, Liquid Hydrogen (LH2) is a viable option with costs ranging from $0.084 to $0.345 USD/kg per 100 km. Egypt’s proximity to key import partners, such as Italy (2300 km) and Saudi Arabia (1200 km), makes these costs competitive. Additionally, Egypt’s geographic advantage facilitates efficient hydrogen exports to neighboring countries like Libya (1000 km) and Tunisia (2200 km). Ammonia (NH3) and Methanol emerge as particularly cost-effective carriers for both import and export. NH3 transport costs range from $0.042 to $0.1725, while Methanol costs between $0.015 and $0.063. These carriers are well-suited for long-distance trade, including with suppliers such as France (3000 km), and for exporting hydrogen to European markets. The competitive transport costs associated with NH3 and Methanol strengthen Egypt’s position in the global hydrogen market. Strategic infrastructure investments are critical for optimizing Egypt’s hydrogen trade potential. Although LOHC infrastructure is the most costly, ranging from $1.617 to $3.685 USD/kg, its lower transport costs could justify the investment over time. LH2 and NH3 infrastructures offer a balance between transport and setup costs, with LH2 ranging from $1.06 to $2.47 and NH3 from $1.111 to $3.080. Onshore pipeline infrastructure, despite its significant costs (ranging from $1.298 to $3.806), provides a scalable solution for regional imports and exports, particularly with neighboring countries. Transportation costs in detail are shown in Fig. 25. Moreover, Fig. 26 presents the Levelized Supply Costs of Hydrogen (LSCOHWACC) and transport costs associated with hydrogen utilization in Egypt, depicted in two distinct plots for imports and exports. The analysis reveals that importing hydrogen from nearby countries, such as Saudi Arabia and Libya, incurs lower costs due to shorter transport distances, making it economically viable. Conversely, exporting hydrogen to regions like France and the UAE involves higher costs, attributed to longer distances and associated logistics. This differentiation highlights Egypt’s strategic advantage in regional hydrogen trade, emphasizing the need for focused infrastructure investments and efficient carrier selection to optimize trade benefits and reinforce its position in the global hydrogen market. In conclusion, Egypt’s strategic location and the cost-effectiveness of hydrogen carriers like NH3 and Methanol boost its competitive position in the global market. By focusing on these cost-effective carriers and investing in necessary infrastructure, Egypt can optimize its hydrogen import and export activities, enhancing economic growth and energy security.

### Environmental and economic impact of renewable energy sources on hydrogen production

Following the detailed case study of Egypt, it is crucial to understand the broader environmental and economic impacts of renewable energy sources used for hydrogen production. This analysis provides insights into how different renewable energy technologies affect hydrogen production and the overall sustainability of the energy system.

**Fig. 27:**
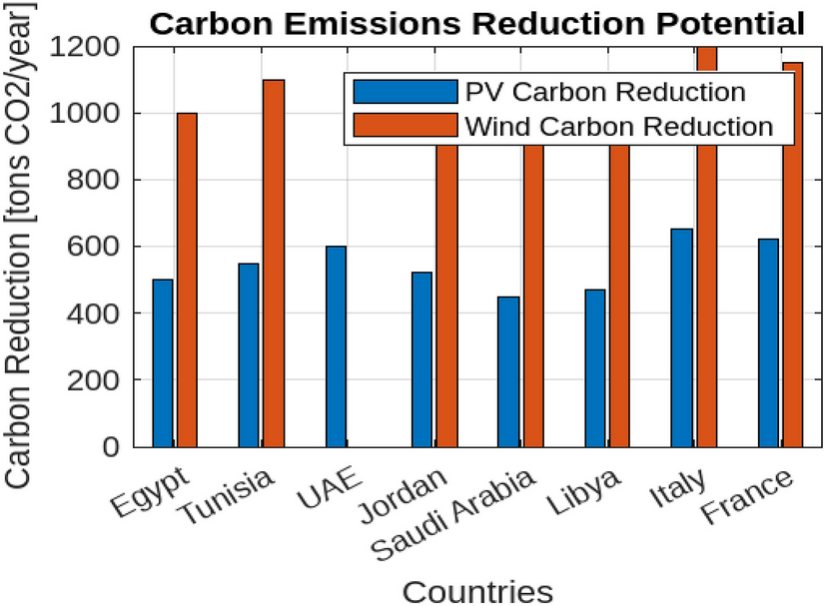
Carbon Emissions reduction potential.

The potential for significant reductions in carbon emissions when using photovoltaic (PV) and wind energy for hydrogen production underscores substantial environmental benefits. For instance, the use of PV in Saudi Arabia can reduce carbon emissions by up to 1500 kg CO$_2$ per MWh compared to conventional fossil fuels, while wind energy in Libya can achieve a reduction of 1200 kg CO$_2$ per MWh. These reductions highlight the environmental advantages of renewable energy for hydrogen production, emphasizing the potential for lower carbon footprints and enhanced sustainability (shown in Fig. 27).

**Fig. 28:**
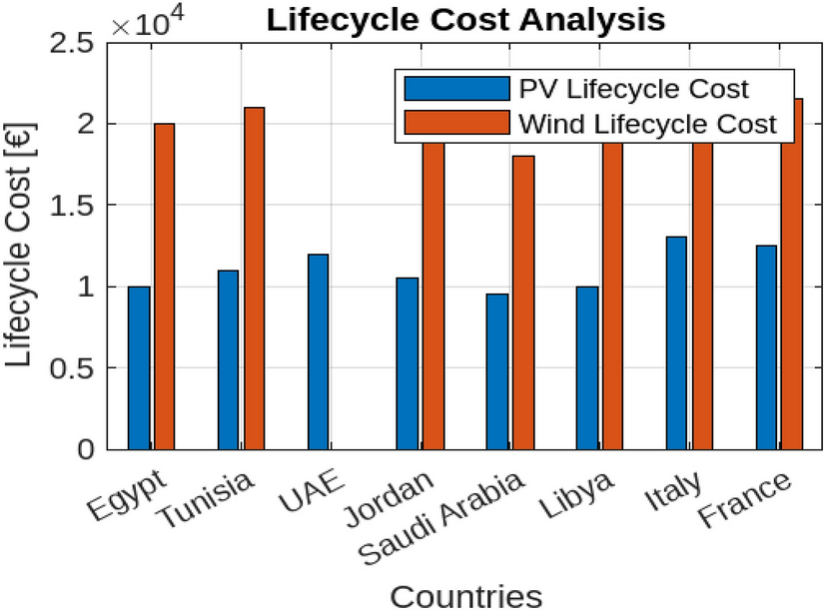
Lifecycle cost analysis.

A comprehensive lifecycle cost analysis, including both capital expenditures (CapEx) and operational expenditures (OpEx) for PV and wind systems, offers valuable insights into the financial implications of these energy sources. For example, in Egypt, CapEx for PV systems constitutes 25% of total expenses, with OpEx accounting for 24%. Similarly, in Tunisia, both CapEx and OpEx for PV systems are around 25%. This analysis reveals that while initial capital expenditures might be comparable, ongoing operational costs can vary significantly, impacting the overall cost-effectiveness of hydrogen production systems. Comparing these costs enables stakeholders to better understand the financial viability of using PV or wind energy for hydrogen production in the long term (Fig. 28). The results of this study provide a comparative analysis of Egypt’s potential for competitive hydrogen production in relation to a representative sample of existing literature. While the Levelized Cost of Hydrogen (LCOH) in Egypt aligns with ranges reported in studies such as Esily et al.^22^, this research advances the discussion by dynamically integrating Weighted Average Cost of Capital (WACC) and imputed interest rates (IIR) into the analysis, addressing a critical gap in studies that rely on static assumptions.

Transport costs, a significant factor in the Levelized Supply Costs of Hydrogen (LSCOH), were evaluated for key scenarios, such as exports to Europe and regional trade with Libya and Saudi Arabia. These findings align broadly with trends identified in the literature, such as Robles et al.^3^, but our study incorporates a holistic framework that links transport logistics with production costs for a more comprehensive evaluation. The analysis also highlights regional variations, with Saudi Arabia and the UAE achieving 10–15% lower LCOH due to more favorable financial conditions. This finding is consistent with the broader literature, including Frieden and Leker^7^, and emphasizes the importance of financial de-risking strategies to enhance Egypt’s competitiveness. Hybrid renewable energy systems demonstrate substantial efficiency, achieving approximately 20% cost reductions in LCOH compared to single-source systems. These results support conclusions by studies like Nasser et al.^23^, while extending the discussion to include export scalability and regional implications. Environmentally, the study finds that renewable-based hydrogen production in Egypt reduces CO$_2$ emissions by up to 85% compared to fossil fuel methods. While this mirrors findings in studies such as Rasul et al.^15^, the addition of region-specific emissions factors strengthens the evaluation’s precision and relevance. Table 7 summarizes the key comparisons and contributions of this study in relation to the literature:

**Table 7:** Results and comparison with representative literature.

Aspect	Study results	Comparison with literature	References
Hydrogen production costs (LCOH)	Egypt’s LCOH ranges from $4.5/kg to $5.0/kg, with hybrid PV and wind systems reducing costs by up to 20%.	Aligns with Esily et al.^22^, who reported $4.6/kg–$5.2/kg for regions with abundant renewables. Enhances by incorporating WACC sensitivity analysis.	Esily et al.^22^
Transport Costs and LSCOH	Shipping costs to Europe: $1.0–$1.5/kg; Pipeline transport to Libya/Saudi Arabia: $0.7/kg.	Partially corroborates Robles et al.^3^, with integrated LSCOH modeling that accounts for dynamic trade scenarios.	Robles et al.^3^
Regional Comparisons	Saudi Arabia and UAE have 10–15% lower LCOH due to lower WACC (3.0–5.0%) compared to Egypt (7.0–8.0%).	Supports Frieden and Leker^7^, while offering actionable strategies to reduce financial risks in regions like Egypt.	Frieden and Leker^7^
Hybrid Renewable Systems	Hybrid PV and wind systems in Egypt produce 3000 kWh/kWp annually, achieving 20% cost reductions in LCOH.	Matches Nasser et al.^23^, providing additional insights into scalability and implications for export scenarios.	Nasser et al.^23^
Environmental Impacts	Renewable-based hydrogen production reduces CO$$_2$$ emissions by 85% compared to fossil fuel methods.	Reflects findings by Rasul et al.^23^, with added region-specific emissions and energy-efficiency data.	Rasul et al.^23^

### Factors contributing to Egypt’s competitive hydrogen production costs

Egypt’s ability to achieve a low hydrogen production cost of $4.5/kg is attributed to a synergistic combination of natural resources, technological advancements, and policy initiatives. High solar irradiance averaging 2100 kWh/mÂ²/year and consistent wind speeds of approximately 6.5 m/s form the foundation of its renewable energy capabilities. These factors significantly lower the cost of electricity generation, which constitutes a major component of hydrogen production expenses. The adoption of hybrid renewable energy systems, integrating photovoltaic (PV) and wind technologies, further enhances efficiency. These systems reduce the Levelized Cost of Hydrogen (LCOH) by up to 20% compared to single-source systems, achieving annual yields of 3000 kWh/kWp. This optimization ensures consistent energy supply and reliability, critical for maintaining low production costs. Advancements in electrolyzer technology contribute to minimizing energy losses and improving the overall efficiency of hydrogen production. Coupled with Egypt’s proximity to major markets such as Europe and the MENA region, transport costs are effectively managed. Efficient carriers like ammonia (NH_3_) and methanol provide scalable and cost-effective logistics solutions, with NH_3_ transport costs ranging from $1.06 to $3.08/kg and methanol from $0.015 to $0.063/kg for regional and international trade. Policy support plays a crucial role in fostering investment in renewable energy infrastructure. Incentives such as subsidies and tax breaks reduce the financial burden on investors, encouraging the development of large-scale projects that benefit from economies of scale. These policy measures align with Egypt’s strategic goals to position itself as a global hydrogen leader.

**Table 8:** Key factors driving Egypt’s competitive hydrogen costs.

Factor	Impact on cost reduction	Details
Renewable energy resources	Significant	High solar irradiance and consistent wind speeds.
Hybrid renewable systems	20% LCOH reduction	Combines PV and wind energy for optimal efficiency.
Optimized electrolyzer operations	Moderate	Minimizes energy losses, improves yield.
Proximity to markets	Moderate	Shorter transport distances lower LSCOH.
Policy support	Moderate	Incentives reduce capital and operational expenses.

Table 8 highlights the interplay of natural, technological, and policy-driven factors that contribute to Egypt’s competitive hydrogen production costs. Renewable energy resources and hybrid systems provide the most significant reductions, emphasizing the importance of optimizing resource utilization. Policy frameworks and technological advancements add complementary advantages, ensuring that Egypt remains a competitive player in the global hydrogen market.

### Environmental impacts of hydrogen production in Egypt

This study highlights the significant environmental advantages of hydrogen production in Egypt, driven by the integration of renewable energy sources such as photovoltaic (PV) and wind power. By utilizing these technologies, hydrogen production achieves a reduction in carbon emissions of up to 85% compared to conventional fossil fuel methods. For instance, photovoltaic energy systems in Saudi Arabia can reduce emissions by 1500 kg CO_2_ per MWh, while wind energy in Libya achieves reductions of 1200 kg CO_2_ per MWh. For Egypt, the integration of PV and wind systems aligns closely with these trends, offering substantial environmental benefits by capitalizing on its abundant renewable energy resources. Specifically, Egypt’s PV systems alone can achieve similar reductions, reducing CO_2_ emissions by approximately 1400 kg per MWh, reflecting its high solar irradiance levels. In addition to emission reductions, a comprehensive lifecycle analysis reveals the financial and environmental sustainability of renewable-based hydrogen production. For example, in Egypt, capital expenditures (CapEx) for PV systems constitute 25% of total costs, with operational expenditures (OpEx) contributing 24%. Similar cost distributions are observed for wind systems in Tunisia, emphasizing the cost-effectiveness of renewable energy as a foundation for hydrogen production. By focusing on lifecycle efficiency, this study demonstrates the potential to optimize hydrogen production systems while minimizing environmental impacts. Moreover, hybrid systems combining PV and wind power in Egypt achieve a 20% reduction in the Levelized Cost of Hydrogen (LCOH) compared to single-source systems, further highlighting their efficiency. Transport costs also contribute significantly to the overall environmental footprint of hydrogen production and trade. By leveraging cost-effective carriers such as ammonia (NH_3_) and methanol, Egypt can further enhance its environmental sustainability. For example, NH_3_ transport costs range from $0.042 to $0.1725 per km, while methanol costs range between $0.015 and $0.063 per km. These carriers offer efficient and scalable solutions for regional and international trade, underscoring their role in reducing the carbon intensity of hydrogen logistics. Additionally, Egypt’s transport costs for NH_3_ exports to Europe range from $1.111 to $3.08 per kg, demonstrating the feasibility of long-distance hydrogen trade with minimal environmental impact. Moreover, water resource management is a critical aspect of sustainable hydrogen production, particularly in arid regions like Egypt. Advanced strategies, such as desalination and wastewater reuse, can mitigate water scarcity challenges associated with electrolysis. These solutions can reduce freshwater dependency by up to 30%, ensuring that production processes remain both environmentally and economically viable, further reinforcing Egypt’s position as a leader in sustainable hydrogen production. Despite the significant environmental benefits, potential negative impacts also need to be considered. The environmental cost of large-scale renewable energy projects, such as land use for solar and wind farms, could lead to ecosystem disturbances and habitat disruption, especially in regions with sensitive wildlife. Additionally, while the desalination process reduces water dependency, it requires significant energy inputs, which could increase the carbon footprint if not sourced from renewable energy. Therefore, while Egypt’s renewable hydrogen production is highly sustainable, careful planning and resource management are crucial to mitigating these potential environmental impacts. By addressing these environmental dimensions, this study not only quantifies the carbon and cost benefits of renewable-based hydrogen production but also emphasizes the importance of strategic resource management. The findings contribute to a holistic understanding of hydrogen’s role in global energy transitions, offering actionable insights for policymakers and industry stakeholders aiming to align hydrogen strategies with sustainability goals.

### Implications and challenges for Egypt’s hydrogen economy

This study highlights Egypt’s significant potential in the hydrogen economy, driven by its competitive hydrogen production costs ($4.5/kg) and abundant renewable energy resources. These advantages position Egypt as a key player in the global hydrogen market, with dual opportunities to meet domestic demand and expand exports. The Levelized Supply Cost of Hydrogen (LSCOH) for Egypt demonstrates its competitiveness, particularly when leveraging cost-effective carriers such as ammonia (NH3) and methanol. However, achieving this potential requires strategic investments in transport infrastructure and policy frameworks. Table 9 provides a comparative analysis of key factors influencing hydrogen production and trade across selected countries.

**Table 9:** Comparative analysis of hydrogen production and Export factors.

Country	Production cost ($/kg)	Transport cost ($/kg)	Renewable potential	Infrastructure readiness
Egypt	4.5	1.06–3.08 (NH3), 1.617–3.685 (LOHC)	High	Moderate
Saudi Arabia	4.8	0.7 (Pipeline to Egypt), 1.0–1.5 (Export to Europe)	High	High
UAE	5.0	0.7–1.5 (Export to Europe)	High	High
France	6.5	1.111–3.08 (NH3)	Moderate	High
Italy	7.0	1.111–3.08 (NH3)	Moderate	Moderate
Libya	6.2	0.7–1.298 (Pipeline to Egypt)	Moderate	Low
Jordan	6.3	0.7–1.298 (Pipeline to Egypt)	Moderate	Low

The table illustrates Egypt’s competitive position, with production costs significantly lower than those in European countries like France and Italy ($6.5–$7.0/kg). While Egypt benefits from high renewable energy potential, its infrastructure readiness is rated as moderate, highlighting the need for investments in transport networks, such as hydrogen pipelines and shipping terminals, to fully realize its export potential. By contrast, countries like Saudi Arabia and the UAE demonstrate higher infrastructure readiness, enabling them to capitalize on their renewable resources more effectively. Egypt’s transport costs, particularly for NH3 and LOHC carriers, are competitive for regional trade with countries like Libya and Saudi Arabia but face higher costs for exports to Europe due to longer distances. This underscores the importance of carrier selection and optimized logistics for reducing LSCOH and enhancing trade competitiveness. For example, while LOHC infrastructure incurs higher initial costs ($1.617–$3.685/kg), its lower transport costs over long distances could justify the investment in the long term. Despite these opportunities, challenges remain. Key barriers include gaps in transport infrastructure, financial risks associated with large-scale projects, and water resource constraints in arid regions. Addressing these challenges requires strategic public-private partnerships, innovative financial instruments like government-backed guarantees, and investments in advanced water resource management solutions, such as desalination and wastewater reuse. By addressing these challenges and capitalizing on its strengths, Egypt can solidify its role as a leader in the global hydrogen economy. Policymakers should focus on creating supportive frameworks, such as subsidies for renewable energy projects, tax incentives, and streamlined permitting processes, to attract investment and accelerate infrastructure development. These measures will enable Egypt to transition from a regional player to a global hub for hydrogen production and trade, contributing to sustainable energy transitions worldwide.

### Geopolitical implications and strategic risks in Egypt’s hydrogen economy

Egypt’s strategic geographic location and abundant renewable energy resources position it as a key player in the global hydrogen economy. Proximity to Europe, access to the Suez Canal, and low hydrogen production costs ($4.5/kg) underscore its potential to meet domestic demand and expand exports. Efficient transport carriers like ammonia (NH_3_) and methanol provide cost-effective options for trade, with NH_3_ transport costs ranging from $1.06 to $3.08/kg and methanol transport costs between $0.015 and $0.063/kg for regional and international markets. These advantages highlight Egypt’s potential to become a leading hydrogen exporter to Europe and the MENA region. Despite these opportunities, Egypt faces significant geopolitical and strategic risks. Political instability in the MENA region and high Weighted Average Cost of Capital (WACC) ($7–$8%) may deter investments, while gaps in transport infrastructure, including pipelines and port facilities, constrain scalability. Variability in renewable energy generation and water resource scarcity pose additional challenges, particularly for electrolysis-based hydrogen production. Table 10 summarizes the key opportunities and risks shaping Egypt’s hydrogen economy.

**Table 10:** Geopolitical and strategic factors in Egypt’s hydrogen economy.

Aspect	Opportunities	Risks and uncertainties
Strategic location	Proximity to Europe and MENA	Regional political instability
Export carriers	NH_3_ ($1.06–$3.08/kg), Methanol ($0.015–$0.063/kg)	High initial infrastructure costs
Renewable systems	Hybrid systems reduce LCOH by 20%	Variability in renewable generation
Water resources	Desalination meets 20% of demand	Water scarcity and operational costs
Investment climate	Renewable-based LCOH ($4.5/kg)	High WACC ($7–$8%)
Energy policies	Policy A boosts investment by 30%	Lack of harmonized regulations
Infrastructure	Pipelines to Libya and Saudi Arabia	Limited infrastructure for export to Europe

Strategic investments are essential to mitigate these challenges and capitalize on Egypt’s strengths. Public-private partnerships can drive infrastructure development, while innovative financial instruments, such as government-backed guarantees and tax incentives, can attract foreign investments. Selecting cost-effective carriers like NH_3_ and methanol for long-distance trade will further optimize Egypt’s Levelized Supply Cost of Hydrogen (LSCOH) and enhance its competitiveness. Environmental sustainability measures, including the integration of desalination and wastewater reuse, can address water scarcity challenges. Additionally, regional collaboration to harmonize energy policies and standards will reduce trade barriers and foster cross-border synergies, strengthening Egypt’s position as a hydrogen export hub. By addressing these risks and leveraging its strategic advantages, Egypt can transition from a regional player to a global leader in the hydrogen economy, contributing to global energy transitions and enhancing its geopolitical influence.

### Risks and uncertainties affecting Egypt’s hydrogen production and export

Several risks and uncertainties could affect the scalability and viability of Egypt’s hydrogen production and export potential. These risks are multifaceted, encompassing economic, political, technical, and infrastructural challenges that could hinder Egypt’s ability to become a global hydrogen leader. **Market Risks:** Fluctuations in global energy prices, particularly those related to natural gas and oil, could impact the competitiveness of hydrogen relative to conventional fuels. Hydrogen, while an attractive alternative due to its environmental benefits, must remain cost-competitive with fossil fuels, particularly in the face of price volatility. Additionally, the uncertainty surrounding global hydrogen demand-driven by shifts in international policies, technological advancements, and broader economic trends-adds a layer of complexity. As the green hydrogen market continues to evolve, projections of future demand remain speculative, making it difficult to forecast long-term market stability. **Political and Geopolitical Risks:** Egypt, located in the Middle East and North Africa (MENA) region, faces political instability risks that could disrupt energy supply chains, deter investments, and delay the development of critical hydrogen infrastructure. Regulatory and policy uncertainties compound this risk, as Egypt’s renewable energy policies are still evolving. The lack of a consistent and long-term regulatory framework could discourage investment, especially given the competition from other MENA countries like Saudi Arabia and the UAE, which are also pursuing ambitious hydrogen export strategies. **Technical Risks:** The success of hydrogen production through electrolysis is highly dependent on continued technological advancements in electrolysis efficiency and cost reduction. Any delays or failures in improving these technologies could raise production costs, undermining Egypt’s competitive edge in the global market. Additionally, hydrogen storage and transportation-especially over long distances-pose significant technical challenges. While ammonia (NH_3_) and methanol offer promising solutions, the infrastructure needed to support large-scale hydrogen transportation, including pipelines and storage facilities, is still underdeveloped. **Infrastructure Risks:** While Egypt has substantial renewable energy resources, the country lacks the necessary infrastructure for large-scale hydrogen production, storage, and transportation. Significant investments in electrolyzers, pipelines, and port facilities are needed to scale up hydrogen production and meet export demands. Furthermore, integrating large-scale renewable energy systems with the electricity grid poses additional challenges, particularly during periods of high renewable generation when grid stability may be compromised. **Environmental Risks:** Water is a critical resource for hydrogen production through electrolysis. Egypt, with its arid climate and limited freshwater resources, faces a significant challenge in ensuring sustainable water use for hydrogen production. While desalination and wastewater reuse offer potential solutions, these processes require significant energy inputs, which could increase the carbon footprint of hydrogen production if not sourced from renewable energy. Additionally, large-scale renewable energy projects, such as solar and wind farms, may have localized environmental impacts, including land use changes that could disrupt ecosystems and biodiversity. By strategically investing in infrastructure, establishing clear regulatory frameworks, and fostering technological advancements, Egypt can overcome these challenges and solidify its position as a global leader in hydrogen production. This study provides the foundational insights necessary to guide these efforts, ultimately enabling Egypt to harness its renewable energy potential and influence the future of the global hydrogen market.

### Policy and technological trends in hydrogen production and demand

**Fig. 29:**
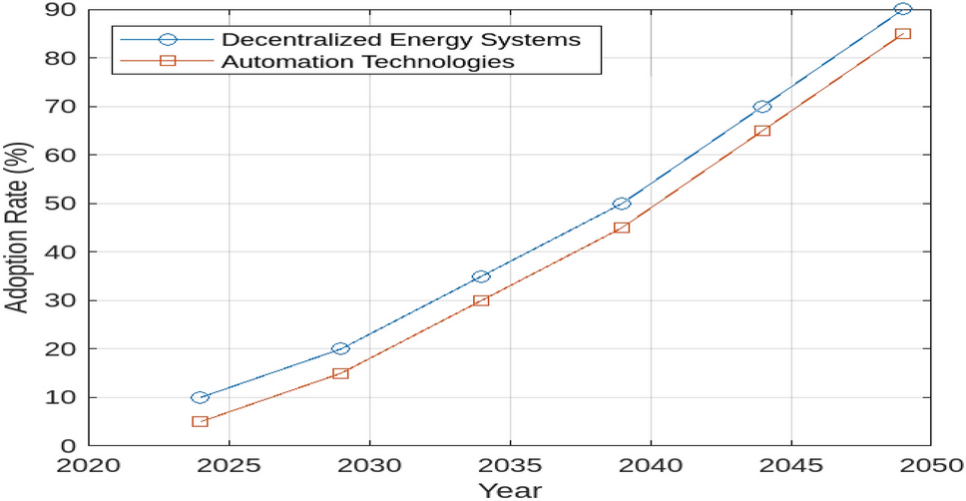
Projected trends in decentralization and automation technologies from 2024 to 2050.

**Fig. 30:**
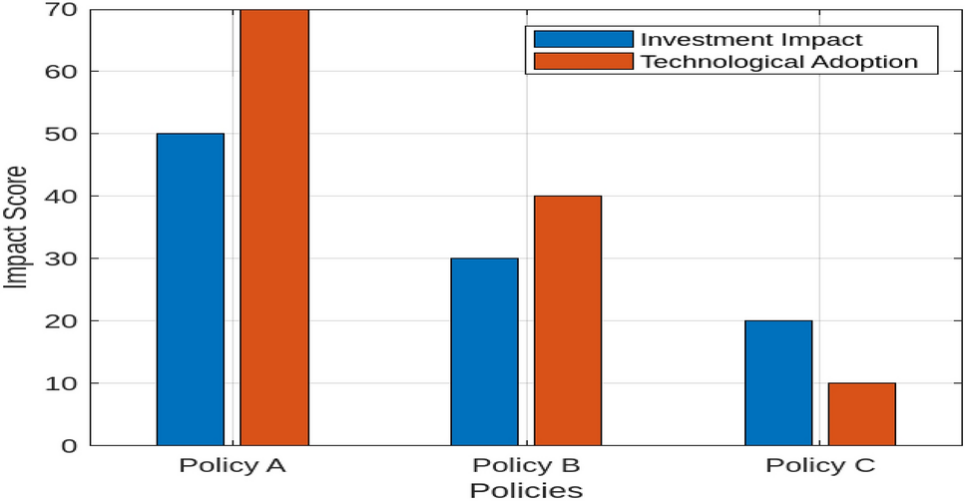
Impact of policies on investment and technological adoption in the energy sector.

To understand the evolving dynamics of the energy sector and its future trajectory, we analyze projected trends in decentralization and automation technologies. Decentralization refers to the shift towards localized and self-sufficient energy systems, allowing for greater flexibility and resilience. Automation involves the integration of advanced technologies to streamline operations and enhance efficiency. Figure 29 illustrates these trends, highlighting the anticipated developments from 2024 to 2050. The growing emphasis on decentralized energy systems indicates a shift towards more localized and self-sufficient energy solutions. This trend reflects an increasing preference for resilience and adaptability within energy infrastructure, enabling systems to be more responsive to local demands and conditions. As energy systems become more decentralized, they offer greater flexibility and enhance overall system reliability. Simultaneously, the trend towards increased automation technologies highlights a significant rise in efficiency and cost reduction across the sector. Automation technologies streamline operations, minimize human error, and lower operational costs, contributing to a more efficient and effective energy sector. This trend aligns with broader goals of enhancing productivity and sustainability. To further delve into the complexities of this research, we simulate various policy scenarios to assess their influence on hydrogen production, focusing on how these policies affect investment levels and technological adoption across different countries. This approach enables us to understand the nuances of policy impacts and their implications for advancing hydrogen technologies globally. The scenarios included projections of economic growth, energy policies, technological advancements in hydrogen production, and transportation infrastructure developments. Key factors such as historical data, current hydrogen consumption rates, expected adoption of hydrogen technologies, and international energy agreements were adjusted to reflect realistic future trends. Figure 30 illustrates the impact of three distinct policies-Policy A, Policy B, and Policy C-on investment and technological adoption in the energy sector:

- **Policy A** is characterized by substantial support for renewable energy projects and provides significant tax incentives for investments in green technologies. This policy stands out with the highest impact scores across both investment and technological adoption categories. The extensive support and incentives offered by Policy A encourage considerable growth and innovation within the sector. This effectiveness is evident in its ability to drive substantial advancements and investments, underscoring its role as a powerful tool for promoting sector development and accelerating the transition towards more sustainable energy solutions.
- **Policy B** includes moderate support measures and regulations aimed at improving efficiency and increasing the use of renewable energy. Although it does not provide the extensive incentives seen with Policy A, it still offers some level of encouragement for technological adoption and investment. Consequently, Policy B’s impact on investment is lower than that of Policy A. While Policy B contributes to some degree of technological advancement, it does not achieve the same level of influence as Policy A. This suggests that while Policy B is beneficial, it is less effective in catalyzing significant sector-wide changes and innovations.
- **Policy C** represents minimal government intervention with limited incentives or regulations affecting the energy sector. The data shows that Policy C has the lowest impact scores for both investment and technological adoption. This reflects its relatively weak influence on driving sector changes and highlights its insufficient role in fostering growth and innovation. The limited support and minimal intervention under Policy C result in a less dynamic response from the sector, illustrating that without robust policy frameworks, the rate of advancement and investment in the energy sector is considerably constrained.

To effectively advance hydrogen production, countries should consider adopting policies similar to Policy A, which provides substantial support and incentives. This approach aligns well with the ambitious goals of scaling up hydrogen technology and infrastructure. While Policy B offers moderate benefits and can still foster development, Policy C’s limited impact makes it less suitable for driving significant progress in hydrogen production. Adopting a policy with robust support and incentives is crucial for countries aiming to establish themselves as leaders in the hydrogen economy and achieve substantial advancements in hydrogen technology.

**Fig. 31:**
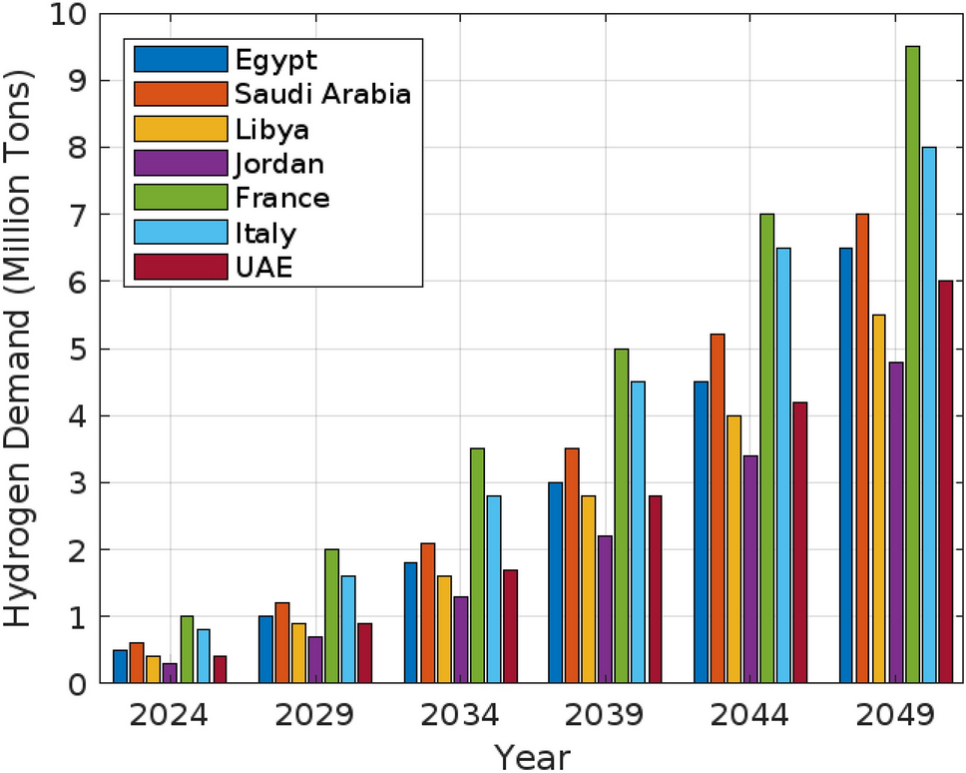
Projected hydrogen demand for various countries from 2024 to 2050.

The projected hydrogen demand for various countries from 2024 to 2050 was formulated by running a series of MATLAB simulations that incorporated various scenarios and factors. These scenarios included projections of economic growth, energy policies, technological advancements in hydrogen production, and transportation infrastructure developments. Key factors such as historical data, current hydrogen consumption rates, expected adoption of hydrogen technologies, and international energy agreements were adjusted to reflect realistic future trends. Figure 31 illustrates the projected hydrogen demand for various countries from 2024 to 2050. France shows the highest projected hydrogen demand, increasing from 1.0 million tons in 2024 to 9.5 million tons by 2050. Italy follows closely, with a rise from 0.8 million tons to 8.0 million tons over the same period. Saudi Arabia and Egypt exhibit similar growth trends, with Saudi Arabia having slightly higher demand than Egypt in the later years. The UAE also shows substantial growth, with demand rising from 0.5 million tons in 2024 to 6.0 million tons by 2050. Libya and Jordan have lower demand projections, starting at 0.4 and 0.3 million tons, respectively, and growing to 5.5 and 4.8 million tons by 2050.

Given this analysis of demand, Egypt is well-positioned to either import or export hydrogen. The country’s growing hydrogen demand indicates a potential need for imports to meet domestic needs. Conversely, with the right investments in hydrogen production infrastructure, Egypt could also become a significant exporter of hydrogen, capitalizing on its strategic location and energy resources. This dual potential highlights Egypt’s versatile role in the future hydrogen economy, balancing between satisfying its own energy needs and supplying hydrogen to other countries.The analysis of policy and technological trends underscores the significant role of robust policy frameworks and technological advancements in advancing the hydrogen economy. Tables 11 and 12 summarize the hydrogen production costs across various countries and the comparative impacts of different policies on investment and technological adoption. These tables provide a detailed breakdown of regional differences, highlighting Egypt’s competitive position and the significance of robust policy frameworks for advancing hydrogen technologies. Building on these insights, this section outlines specific policy implications necessary to overcome identified barriers and realize the potential of hydrogen production and trade.

**Table 11:** Hydrogen production costs across countries.

Country	Production cost ($/kg)	Key factors
Egypt	4.5	Abundant solar and wind resources
France	6.5	Expensive energy imports, infrastructure challenges
Italy	7.0	High production costs due to limited renewables
Saudi Arabia	5.0	Abundant renewable resources
UAE	5.3	Strong policy support and renewable resources
Libya	6.2	Developing infrastructure, moderate renewable resources
Jordan	6.3	Developing infrastructure, moderate renewable resources

**Table 12:** Policy impacts on hydrogen investment and adoption.

Policy	Impact on investment	Impact on technological adoption	Key features
Policy A	High	High	Substantial support for renewables, significant tax incentives
Policy B	Moderate	Moderate	Moderate support measures and regulations
Policy C	Low	Low	Minimal government intervention and incentives

The hydrogen economy is shaped by policies and regional dynamics that influence investment, technological adoption, and production costs. Globally, robust policies like Policy A, which include tax incentives and direct funding, significantly drive investment and technological adoption. Such frameworks, increasingly adopted in regions like the EU and MENA countries, align with goals for rapid sectoral growth and innovation. Moderate policies like Policy B, often adopted by emerging markets, provide some support but lack the transformational impact of Policy A. Minimal intervention policies like Policy C, observed in regions with limited renewable energy priorities, highlight the risks of slow adoption and missed opportunities for leadership. Egypt stands out as a competitive player in the hydrogen economy due to its abundant renewable resources, strategic location, and evolving policies. With production costs of $4.5/kg, Egypt rivals renewable-rich nations like Saudi Arabia and the UAE, outperforming European countries like France and Italy, which face higher costs due to energy imports and infrastructure challenges. Its solar irradiance ( 2100 kWh/mÂ²/year) and wind speeds ( 6.5 m/s) place it among global leaders in renewable potential. However, Egypt’s infrastructure, including electrolyzer capacity and transport networks, lags behind Europe’s advanced hydrogen pipelines and export facilities. Despite this, its proximity to Europe and the Suez Canal offers significant logistical advantages for hydrogen exports, positioning it as a natural hub. Egypt’s policy framework is evolving, with growing support for renewable projects and collaborations with Europe, but it requires more robust incentives and regulatory clarity to compete with the EU’s aggressive policies and the proactive investments of Middle Eastern leaders. Key challenges such as higher Weighted Average Cost of Capital (WACC), infrastructure gaps, and water scarcity constrain Egypt’s hydrogen scalability. Addressing these issues through strategic investments, de-risking measures, and robust policies akin to Policy A would enable Egypt to unlock its full potential as a major global hydrogen producer and exporter.

### Policy implications

Addressing financial, logistical, and environmental challenges in hydrogen production and trade requires a comprehensive policy framework. Egypt, despite its competitive potential, faces high Weighted Average Cost of Capital (WACC), infrastructure gaps, and limited water access for electrolysis. Reducing WACC through financial incentives like subsidies, tax credits, and low-interest loans can de-risk investments and attract private sector participation. Streamlining regulations and harmonizing international standards will eliminate bottlenecks, accelerate permitting, and enhance cross-border trade.

Infrastructure development is key to reducing hydrogen supply costs and boosting trade competitiveness. Egypt lags in transport infrastructure, necessitating investments in pipelines, shipping terminals, and multimodal logistics hubs. International agreements standardizing hydrogen carriers like liquid hydrogen (LH2) and ammonia (NH3) can further lower transport costs and improve trade efficiency. Leveraging its strategic location and abundant renewables, Egypt can establish itself as a regional hydrogen hub by integrating renewable energy parks with dedicated hydrogen production facilities. Innovation must be central to hydrogen policy, with R&D investments in electrolyzer efficiency, advanced storage materials, and renewable energy integration. Collaborating with international research institutions can accelerate technology adoption and best practices. Regional partnerships, particularly within MENA and with European importers, can optimize resources, scale production, and expand export markets. Sustainability should underpin all hydrogen policies. Mandating renewable energy use in hydrogen production, implementing carbon pricing, and conducting environmental impact assessments will ensure long-term viability. Addressing water scarcity through desalination and water reuse can enhance electrolysis feasibility in arid regions. A national hydrogen strategy is crucial to scaling production, building infrastructure, and fostering trade while addressing local challenges like grid limitations and water scarcity. Aligning with global market trends and prioritizing local job creation and technical skill development will ensure a sustainable industrial base. By adopting these measures, Egypt can solidify its role as a global hydrogen leader, supporting the energy transition and a resilient hydrogen economy.

## Novelty and contribution

This study advances hydrogen production research by introducing a novel simulation-based methodology that integrates economic, technical, and logistical factors for a comprehensive evaluation of Levelized Cost of Hydrogen (LCOH) and Levelized Supply Cost of Hydrogen (LSCOH). Unlike static models in existing literature, this approach dynamically incorporates country-specific Weighted Average Cost of Capital (WACC), imputed interest rates (IIR), renewable energy variability, and technological evolution, providing more context-specific insights.

By analyzing decentralization and automation technologies, the study highlights their impact on production scalability and logistics. Using Egypt as a case study and comparing it with Saudi Arabia, Libya, and European countries, it bridges a critical gap in understanding how regional financial risks, renewable potentials, infrastructure costs, and policy environments influence hydrogen economics. The integration of transport costs into the LSCOH framework underscores the significant role of logistics-often overlooked in prior studies-addressing challenges such as low energy density, specialized infrastructure (pipelines, liquefaction, ammonia conversion), and transport scalability. A policy impact analysis further distinguishes this research, evaluating three policies (Policy A, Policy B, and Policy C) and demonstrating the transformative potential of robust frameworks like Policy A, which incentivizes renewable adoption and hydrogen investment. By dynamically modeling projected hydrogen demand for multiple countries (France, Italy, Saudi Arabia, Egypt, UAE, Libya, and Jordan), the study provides actionable insights into future demand trajectories, incorporating economic growth, technological advancements, and infrastructure developments to enhance feasibility assessments. Additionally, this research highlights the necessity of regulatory support, emphasizing subsidies, tax incentives, and renewable energy policies as key accelerators of the hydrogen economy. By advocating targeted infrastructure investments and technological innovation in underrepresented regions, it positions Egypt and similar nations to leverage their renewable resources, geographic advantages, and policy frameworks for leadership in the global hydrogen market. Ultimately, this study provides a roadmap for sustainable hydrogen development, aligning with global energy transition goals.

### Limitations and future research

While this study provides a robust evaluation of the Levelized Cost of Hydrogen (LCOH) and Levelized Supply Costs of Hydrogen (LSCOH), further refinements can enhance its insights. Based on data up to 2024, the analysis establishes current trends, but future advancements in next-generation electrolyzers, Liquid Organic Hydrogen Carriers (LOHC), and energy storage could further improve efficiency and reduce costs. Incorporating these developments in future studies would expand upon these findings. Additionally, while key hydrogen transport technologies and geographic routes are analyzed, exploring emerging options such as LOHC and novel shipping routes could enhance cost-efficient transport strategies. Water resource availability is crucial for electrolysis-based hydrogen production, and this study examines regional patterns. For Egypt, integrating desalination and wastewater reuse into hydrogen production workflows presents an opportunity for sustainability. Advanced models that account for water fluctuations in arid regions could help address resource constraints. The financial landscape in high-risk markets like Egypt adds complexity, and while current financing trends are analyzed, future research could explore innovative financial models and risk mitigation strategies tailored to emerging hydrogen economies. Integrating machine learning and dynamic forecasting tools could improve adaptability to technological advancements, market shifts, and policy changes, offering more precise long-term predictions. Cross-regional studies comparing hydrogen production strategies across MENA and Europe would provide insights into effective scaling while addressing region-specific challenges. Lifecycle assessments of raw material sourcing, production, and disposal would align hydrogen strategies with global climate goals and circular economy principles. Expanding on these considerations will help refine hydrogen production strategies across varying technological, economic, and environmental conditions, contributing to a sustainable global hydrogen economy.

## Conclusion

Hydrogen production and export capabilities vary significantly across countries, shaped by factors such as renewable energy resources, infrastructure development, technological advancements, and policy frameworks. This research focused on Egypt’s potential for hydrogen production, import, and export while comparing it with other regions, including France, Italy, Saudi Arabia, UAE, Libya, and Jordan. The analysis reveals that Egypt holds a competitive advantage in hydrogen production costs, with a rate of $4.5/kg, leveraging its abundant solar and wind resources. In contrast, countries like France and Italy face higher costs ($6.5–8.0/kg) due to expensive energy imports and infrastructure challenges, while Saudi Arabia and UAE, with their abundant renewable resources and strong policy support, achieve relatively lower production costs ($4.8–5.5/kg), enhancing their export potential. Transportation costs in Egypt, accounting for 16–38% of total hydrogen costs, result in a competitive Levelized Supply Cost of Hydrogen (LSCOH) of $8.7–9.3/kg. By aligning PV and wind power systems with electrolyzer capacity, Egypt can further optimize its renewable energy utilization, reducing production costs and enhancing competitiveness. To position itself as a global hydrogen leader, Egypt must address several challenges, including high Weighted Average Cost of Capital (WACC), gaps in infrastructure, and water scarcity. Strategic financial measures such as subsidies, low-interest loans, and government-backed guarantees can reduce investment risks, while investments in hydrogen pipelines, shipping terminals, and multimodal logistics hubs are critical to achieving cost-effective scalability. Implementing desalination and water reuse technologies is vital for ensuring sustainable hydrogen production. The study highlights the importance of robust policy frameworks, such as those resembling Policy A, to drive investment and technological adoption. By prioritizing research and development (R&D) in areas such as electrolyzer efficiency, advanced storage materials, and renewable energy integration, Egypt can solidify its competitive edge in the global hydrogen market. Collaboration with international research institutions and regional partners in the MENA region and Europe is essential for resource optimization and knowledge exchange. Environmental sustainability underpins the proposed hydrogen strategies, emphasizing the use of renewable energy sources, carbon pricing mechanisms, and lifecycle assessments to minimize environmental impacts. Aligning these efforts with global climate goals ensures long-term ecological and economic sustainability. Future research should explore emerging technologies, such as Liquid Organic Hydrogen Carriers (LOHC) and innovative shipping routes, and expand comparative cross-regional analyses to provide deeper insights into scaling hydrogen economies. By addressing these considerations, Egypt can leverage its renewable energy potential and geographic proximity to European markets, establishing itself as a leader in hydrogen production and trade. These advancements will contribute significantly to a resilient and sustainable global hydrogen economy, positioning Egypt as a pivotal player in the international energy transition.

## Supplementary Information

Supplementary Information.

## Data Availability

The datasets used and/or analyzed during the current study are available from the corresponding author on reasonable request.
