# Supplementary material for: Economic and technical analysis of hydrogen production and transport: a case study of Egypt
Source: Sci Rep. 2025 Mar 15;15:9002. doi: 10.1038/s41598-025-91589-6 (PMC11910559; doi:10.1038/s41598-025-91589-6)
Supplement: Supplementary file 1 — Supplementary Information. [file 41598_2025_91589_MOESM1_ESM.pdf]

1 Annex

1.1 Regional Renewable Energy Performance Metrics

To assess the renewable energy potential for various countries, we conducted detailed calculations for photovoltaic (PV) and wind energy generation. This analysis is crucial for understanding regional capabilities and guiding strategic investments in renewable energy infrastructure. The photovoltaic energy yield is calculated using the equation:

$$E_{\text{PV}} = A \times G \times \eta \tag{1}$$

where E_{PV} is the annual energy yield (kWh), A is the area of the PV panels (m^2), G is the average solar irradiance (kWh/m^2/year), and η is the efficiency of the PV panels. For example, in Egypt, with a solar irradiance of 2100 kWh/m^2/year and an efficiency of 18%, the annual energy yield per 500 m^2 of PV panels would be 189,000 kWh.

For wind energy, the energy yield is determined using:

$$E_{\text{wind}} = \frac{1}{2} \times \rho \times A \times v^3 \times C_p \times T \tag{2}$$

where E_{wind} is the annual energy yield (kWh), ρ is the air density (kg/m^3), A is the swept area of the wind turbine blades (m^2), v is the average wind speed (m/s), C_p is the power coefficient of the wind turbine, and T is the total number of operational hours in a year. For instance, with an average wind speed of 6.5 m/s, a swept area of 5026.5 m^2, and a power coefficient of 0.35, the annual energy yield in Egypt would be approximately 7,735,452 kWh. To ensure precise wind energy calculations, we drew data from several sources, detailed in Table 13. This table presents average wind speeds measured at 10 meters above ground level, along with the calculated energy yields from platforms including PVGIS, RENEWABLES, and the Global Wind Atlas, covering the years 2019 through 2022. The alignment of wind speed data across these platforms underpins the reliability of our energy yield estimates. This analysis, formulated by running a series of simulations, incorporated various scenarios and factors. These scenarios included projections of economic growth, energy policies, technological advancements in hydrogen production, and transportation infrastructure developments. Key factors such as historical data, current hydrogen consumption rates, expected adoption of hydrogen technologies, and international energy agreements were adjusted to reflect realistic future trends for Egypt and other regions.

1.2 Assessment of the Weighted Average Cost of Capital (WACC)

In evaluating the financial viability of energy projects, the Weighted Average Cost of Capital (WACC) serves as a critical metric, providing insight into the average rate of return required by investors based on the company's capital

structure. This comprehensive measure integrates various financial components—equity and debt—by calculating their respective costs and weighting them accordingly.

To compute the WACC, we first determine the costs associated with equity and debt. The cost of equity is calculated using the Capital Asset Pricing Model (CAPM), which incorporates macroeconomic conditions and country-specific risks. The CAPM formula for the cost of equity is:

$$r_e = r_f + \beta \times (r_m - r_f) \tag{3}$$

Here, r_e denotes the cost of equity, reflecting the return expected by equity investors. r_f represents the risk-free rate, which is the return on a risk-free investment such as government bonds. The beta coefficient β measures the stock's volatility relative to the overall market, providing insight into investment risk compared to market fluctuations. Lastly, r_m represents the expected market return, reflecting the overall anticipated return from the market. This equation is essential for incorporating systematic risk and market conditions into the cost of equity calculation, allowing for a more precise assessment of investment risk and potential returns.

The cost of debt is determined by considering the yield to maturity on existing debt or the cost of new borrowing, adjusted for tax savings:

$$r_d = i_d \times (1 - T) \tag{4}$$

In this formula, r_d is the after-tax cost of debt, accounting for the tax shield provided by interest expenses. i_d represents the pre-tax cost of debt, which is the nominal interest rate on the debt, and T is the corporate tax rate, reflecting the proportion of income paid as tax. This adjustment is crucial for providing an accurate representation of the actual cost of borrowing after accounting for tax benefits.

Combining these components, the WACC is calculated using:

$$\text{WACC} = \left(\frac{E}{V} \times r_e\right) + \left(\frac{D}{V} \times r_d\right) \tag{5}$$

In this formula, E denotes the market value of equity, D represents the market value of debt, and V is the total market value of equity and debt ($E+D$). r_e is the cost of equity, and r_d is the after-tax cost of debt. This equation provides a comprehensive measure of the average return required by all investors.

To account for inflation and reflect real values, the WACC can be adjusted using:

$$\text{WACC}_{\text{real}} = \frac{1 + \text{WACC}_{\text{nominal}}}{1 + \text{Inflation Rate}} - 1 \tag{6}$$

Here, $\text{WACC}_{\text{real}}$ represents the inflation-adjusted WACC, reflecting the real cost of capital after accounting for inflation. $\text{WACC}_{\text{nominal}}$ is the nominal WACC, and Inflation Rate denotes the expected rate of inflation. This adjustment ensures a more accurate assessment of the real cost of capital, incorporating the

effects of inflation into investment decisions. Using these formulas, we evaluated the WACC for various countries, considering their unique economic conditions and risks. This approach provided a detailed assessment of the financial feasibility of energy projects, offering a nuanced understanding of investment requirements across different regional contexts.

Table 1: Mean Wind Speed and Calculated Energy Yield

Platform	Year	Mean Wind Speed (10m) [m/s]	Calculated Energy Yield [kW
PVGIS	2019	3.60	1950
	2020	3.65	2000
	2021	3.70	2050
	2022	3.75	2100
RENEWABLES	2019	5.15	4150
	2020	5.20	4200
	2021	5.25	4250
	2022	5.30	4300
GLOBAL WIND ATLAS	2019	5.8	3200
	2020	5.8	3250
	2021	5.8	3300
	2022	5.8	3350
